# Bright NIR-Emitting Styryl Pyridinium Dyes with Large Stokes’ Shift for Sensing Applications

**DOI:** 10.3390/bios13080799

**Published:** 2023-08-09

**Authors:** Nirasha I. Wickramasinghe, Brian Corbin, Devni Y. Kanakarathna, Yi Pang, Chathura S. Abeywickrama, Kaveesha J. Wijesinghe

**Affiliations:** 1 Department of Chemistry, Faculty of Science, University of Colombo, Colombo 00300, Sri Lanka; 2 Department of Chemistry, The University of Akron, Akron, OH 44325, USA

**Keywords:** styryl dyes, pyridinium, regio-effect, near-infrared dyes, fluorescence microscopy, donor-π-acceptor dyes, intramolecular charge transfer (ICT), Stokes’ shift

## Abstract

Two NIR-emitting donor-π-acceptor (D-π-A) type regioisomeric styryl pyridinium dyes (**1a**–**1b**) were synthesized and studied for their photophysical performance and environment sensitivity. The two regioisomers, **1a** and **1b**, exhibited interesting photophysical properties including, longer wavelength excitation (λ_ex_ ≈ 530–560 nm), bright near-infrared emission (λ_em_ ≈ 690–720 nm), high-fluorescence quantum yields (ϕ_fl_ ≈ 0.24–0.72) large Stokes’ shift (∆λ ≈ 150–240 nm) and high-environmental sensitivity. Probe’s photophysical properties were studied in different environmental conditions such as polarity, viscosity, temperature, and concentration. Probes (**1a**–**1b**) exhibited noticeable changes in absorbance, emission and Stokes’ shift while responding to the changes in physical environment. Probe **1b** exhibited a significant bathochromic shift in optical spectra (∆λ ≈ 20–40 nm) compared to its isomer **1a**, due to the regio-effect. Probes (**1a**–**1b**) exhibited an excellent ability to visualize bacteria (*Bacillus megaterium*, *Escherichia coli*), and yeast (*Saccharomyces cerevisiae*) via fluorescence microscopy.

## 1. Introduction

**Scheme 1:**
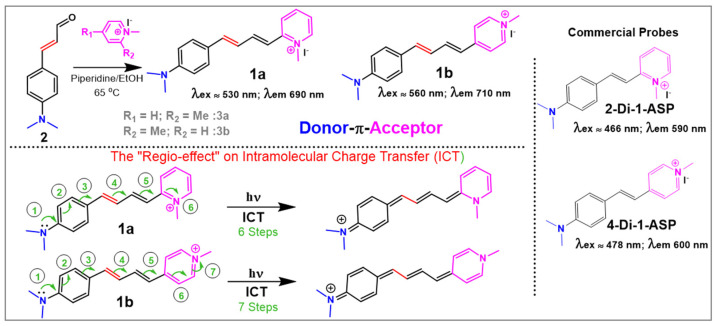
Synthesis of probes **1a**-**1b** and ICT in probe **1a**.

Styryl pyridinium dyes are an important class of conjugated small-molecules for developing efficient fluorescent imaging probes, laser dyes and photosensitizers [1,2,3,4,5,6,7]. The incorporation of the positively charged pyridinium moiety in to the conjugated π-system confers unique photophysical properties to these probes [8]. Donor-π-acceptor (D-π-A) molecules with advantageous photophysical properties can be formed by connecting a moderate to strong electron donating group (i.e., -OR -NR_2_) into the conjugated π-system [9,10,11,12]. Such styryl pyridinium dyes (D-π-A) exhibit large Stokes’ shift, high-fluorescence-quantum yields, improved photostability, high biocompatibility, enhanced solubility, and longer wavelength spectral profiles (i.e., far-red to near-infrared regions) [9,13,14,15]. Styryl pyridinium probes have been successfully exploited for several unique fluorescence imaging applications, including plasma membrane staining, mitochondria imaging and membrane potential determination, neuromast imaging in tissues and nucleus/DNA visualization [7,11,14,16,17,18,19,20,21]. The chemical stability, structural simplicity and bright longer-wavelength emission profiles of these probes are highly advantageous during fluorescence microscopy imaging. By incorporating a strong donor group (-NR_2_) in to the probe architecture, interesting D-π-A type pyridinium probes were developed with a strong intramolecular charge transfer (ICT) ability, which produce large Stokes’ shifts (i.e., ∆λ ≈ 50–200 nm) (Scheme 1) [9]. Such improved Stokes’ shift can be highly beneficial for fluorescence microscopy imaging, as they can significantly reduce background interferences, improve penetration depth and mitigate self-quenching artifacts [9,22]. In addition, ICT-based D-π-A type systems exhibit distinguishable optical responses (i.e., shift in emission/absorption maxima, increase/decrease in fluorescence intensity, ON/OFF in fluorescence, etc.) towards environmental changes that makes them ideal fluorescent sensing molecules [23,24,25,26,27]. Incorporating this principle in to action, several ratiometric, intensiometric and hybrid sensing probes were developed [23,24,25,26,27]. 

2-Di-1-ASP and 4-Di-1-ASP (Scheme 1) are widely used and commercially available red-emitting (λ_ex_ ≈ 466–475 nm; λ_em_ ≈ 580–605 nm) D-π-A type pyridinium dyes that have several important applications in fluorescence microscopy imaging, including mitochondria visualization and membrane potential determination, plasma membrane staining, DNA staining and neuromast imaging in eukaryotic tissues (Scheme 1) [4,28,29]. In order to achieve high tissue penetrability and high biocompatibility, it is desirable to develop far-red to near-infrared (NIR) emitting dyes with longer excitation wavelength profiles and higher photostability [9,30,31,32,33,34]. One possible way to achieve NIR emission by extending conjugation length of the π-spacer group in D-π-A type architecture [9,35]. Inspired by this strategy, analogues with extended conjugation to 2-Di-1-ASP and 4-Di-1-ASP (i.e., **1a** and **1b** in Scheme 1) were reported with NIR-emission profiles (λ_ex_ ≈ 530–560 nm; λ_em_ ≈ 690–720 nm) [36,37,38]. Followed by previous reports [36,37,38], we have continued the study of photophysical performance and biosensing applications of probes **1a**–**1b** in this work. The key features of styryl pyridinium dyes **1a** and **1b** include: (1) bright NIR emission (ϕ_fl_ ≈ 0.24–0.72; λ_em_ ≈ 690–720 nm), (2) high-environmental sensitivity and (3) mega Stokes’ shift (i.e., ∆λ ≈ 150–240 nm). In addition, probes **1a**–**1b** were excellent candidates to visualize microorganisms, such as *Bacillus megaterium*, *Escherichia coli*, and *Saccharomyces cerevisiae*, via widefield fluorescence microscopy. Probe **1** was also found to be an efficient fluorescent sensing candidate for quantifying albumin in aqueous environments with higher accuracy and lower detection limits. 

## 2. Materials and Methods

All chemicals for synthesis were purchased from Acros Organics and VWR and used as they were received without further purification. Molecular biology grade reagents for bacterial cell culture and fluorescence microscopy imaging experiments were purchased from Thermo Fisher, Waltham, USA). NMR characterization data were acquired by Varian 500 MHz NMR spectrometer in deuterated DMSO-*d6*. High-resolution mass spectrometry (HRMS) data were acquired by an ESI-TOF MS system (Waters, Milford, MA, USA). UV-Vis studies were carried out in GENESYS 10S (Thermo Scientific, Waltham, MA, USA) and a Hewlett Packard-8453 (Agilent Technologies, Santa Clara, CA, USA) diode array spectrophotometers at 25 °C. Fluorescence spectroscopy studies were performed in HITACHI F7000 fluorescence spectrophotometer and a HORIBA Fluoromax-4 spectrofluorometer. Fluorescence lifetimes of the dyes were measured by using a time-correlated single-photon counting (TCSPC) method on a Horiba DeltaPro lifetime system, which measures the lifetime range of 30 ps–1 s. This instrument is equipped with a picosecond photon detection module comprising a fast, cooled, photomultiplier with 230–850 nm response. All measurements were performed by exciting the sample solutions with a Horiba DeltaDiode^TM^ DD-485 Laser (peak wavelength at 485 nm +/− 10 nm). Fluorescence microscopy imaging was performed in an Olympus BX53 inverted microscopy system with 100× and 40× magnification. Probes **1a**–**1b** were synthesized according to the previously reported procedure with modifications as reported in the methodology section [7,39]. 

### 2.1. General Procedure for Synthesis

In a 10 mL round-bottom flask, 0.5 mmol of the 4-(Dimethylamino)cinnamaldehyde (**2**) was dissolved by adding 5 mL of anhydrous ethanol at room temperature with continuous stirring. To this bright orange solution, the corresponding methyl pyridinium salt (**3**) (0.5 mmol) was added followed by the addition of piperidine (0.5 mmol) with continuous stirring. The resulting dark-red solution was heated up with continuous stirring at 65 °C for 6 h. Upon completion of the reaction (checked by TLC), the dark-red colored reaction mixture was cooled down to room temperature and poured in to a 100 mL flask pre-filled with ethyl acetate (20 mL) while stirring continuously. The resulting dark red colored precipitate was collected by vacuum filtration and further washed with ethyl acetate (2 × 20 mL) and dried under the vacuum. The product was further purified with 5% methanol in dichloromethane by flash chromatography and dried under high vacuum.

*2-((1E,3E)-4-(4-(dimethylamino)phenyl)buta-1,3-dien-1-yl)-1-methylpyridin-1-ium iodide* (**1a**): Obtained as a bright red solid with 80% isolated yield. ^1^H NMR (500 MHz, DMSO-*d6*) δ 8.78 (dd, J = 6.4, 1.4 Hz, 1H), 8.40 (dd, J = 8.6, 1.6 Hz, 1H), 8.38–8.32 (m, 1H), 7.82 (dd, J = 15.0, 9.7 Hz, 1H), 7.74 (ddd, J = 7.6, 6.3, 1.6 Hz, 1H), 7.51–7.44 (m, 2H), 7.19–7.00 (m, 2H), 6.96 (d, J = 14.9 Hz, 1H), 6.78–6.72 (m, 2H), 4.23 (s, 3H), 3.00 (s, 6H). ^13^C NMR (126 MHz, DMSO-*d6*) δ 152.98, 151.72, 145.97, 145.56, 143.74, 143.26, 129.64, 124.14, 123.96, 123.77, 123.49, 117.23, 112.55, 56.49, 46.02. HRMS (TOF MS ES^+^) found (*m*/*z*) for [M^+^] 265.1706 [C_18_H_21_N_2_^+^]. HRMS calculated found (*m*/*z*) for [M^+^] 265.1699 [C_18_H_21_N_2_^+^]. 

*4-((1E,3E)-4-(4-(dimethylamino)phenyl)buta-1,3-dien-1-yl)-1-methylpyridin-1-ium iodide* (**1b**): Obtained as a dark red solid with 75% isolated yield. ^1^H NMR (500 MHz, DMSO-*d6*) δ 8.63 (d, J = 6.5 Hz, 2H), 7.96 (d, J = 6.4 Hz, 2H), 7.73 (dt, J = 15.3, 5.0 Hz, 1H), 7.40 (d, J = 8.5 Hz, 2H), 6.94 (d, J = 5.1 Hz, 2H), 6.69–6.63 (m, 3H), 4.12 (s, 3H), 2.92 (s, 6H). ^13^C NMR (126 MHz, DMSO-*d6*) δ 153.21, 151.59, 144.97, 143.73, 142.49, 129.56, 124.00, 123.73, 123.54, 122.88, 112.50, 56.49, 46.96. HRMS (TOF MS ES^+^) found (*m*/*z*) for [M^+^] 265.1696 [C_18_H_21_N_2_^+^]. HRMS calculated found (*m*/*z*) for [M^+^] 265.1699 [C_18_H_21_N_2_^+^]. 

### 2.2. Fluorescence Quantum Yields Calculation and Photophysical Property Evaluation

Stock solutions of the probes (**1a**–**1b**) were prepared in spectroscopic grade DMSO at 10 mM concentration. For all spectroscopic studies (absorbance and emission), the working concentration of the probe was 1 × 10^−5^ M unless otherwise specified. While acquiring the emission spectra in different solvents, probes **1a** and **1b** were excited at 530 nm and the emissions were collected from 550 nm to 800 nm (unless otherwise specified). All spectroscopic studies were conducted in spectroscopic grade or double-distilled organic solvents. As all the fluorometric analysis data were acquired in diluted solutions (≈1 × 10^−5^ M or less), the collected spectra were not corrected for the inner filter-effects, assuming it is negligible [40,41,42]. 

The relative fluorescence quantum yields (ϕ_fl_) for probes (**1a**–**1b**) were calculated by using Rhodamine 6G as the standard (in ethanol), where the fluorescence quantum yield of Rhodamine 6G is 0.95 in ethanol. The following equation was used for the fluorescence quantum yield determination at 530 nm [43,44]**.**

(ϕ_fl_)_sample =_ ϕ_ref_ × (*A*_ref_/*A*_sample_) × [(*I*_sample_)/(*I*_ref_)] × (*ɳ*_sample_)^2^/(*ɳ*_ref_)^2^

where *A* is the absorbance of the sample, *I* is the integrated fluorescence intensity and *ɳ* is the refractive index of the solvent. 

### 2.3. Fluorescence Microscopy Imaging

Two bacterial species (*Bacillus megaterium* and *Escherichia coli*) and a unicellular fungal species, *Saccharomyces cerevisiae*, were freshly cultured for fluorescence microscopy imaging experiments. Microorganisms were stained with 5 µM, 10 µM and 20 µM probe concentrations formed by diluting 1 µL of the stock solutions (made in DMSO) of the probes in aqueous media. *Bacillus megaterium* (Gram-positive) and *Escherichia coli* (Gram-negative) were grown in Luria broth (LB) agar plates. A sterilized loop was used to inoculate a single colony of bacteria from a streak plate into microcentrifuge tubes containing the appropriate concentration of the probe **1a** or **1b** in sterile water and incubated for 30 min at room temperature. After staining, a 4 µL culture volume was spotted on a glass slide and secured using a cover slip and the stained bacteria was then imaged by using a fluorescence microscope under 40× and 100× (oil) magnification. Images were acquired by exciting the stained specimen with 532 nm laser line with the standard Cy3 filter settings (580–620 nm) for the emission collection. *Saccharomyces cerevisiae* was grown in sterilized 250 mL conical flask by introducing 0.5 g of the baker’s yeast in to a 100 mL sterile water for 2 h at 37 °C in a rotary shaker. Form this initial culture, 20 µL volume was transferred into a sterile eppendroff tube and staining solution (probe **1a** or **1b**) was introduced to make a final staining concentration of 5 µM, 10 µM or 20 µM. The resulting yeast suspensions were incubated for 30 min at room temperature. Followed by the incubation, 10 µL from each sample was mounted on glass slide, secured with cover slip and imaged by the fluorescence microscope under 40× and 100× magnification. Images were acquired by exciting stained specimens with 532 nm laser line with standard Cy3 filter settings (580–620 nm) for the emission collection. The bright-field images were acquired for all staining experiments.

## 3. Results

### 3.1. Optical Properties

**Figure 1:**
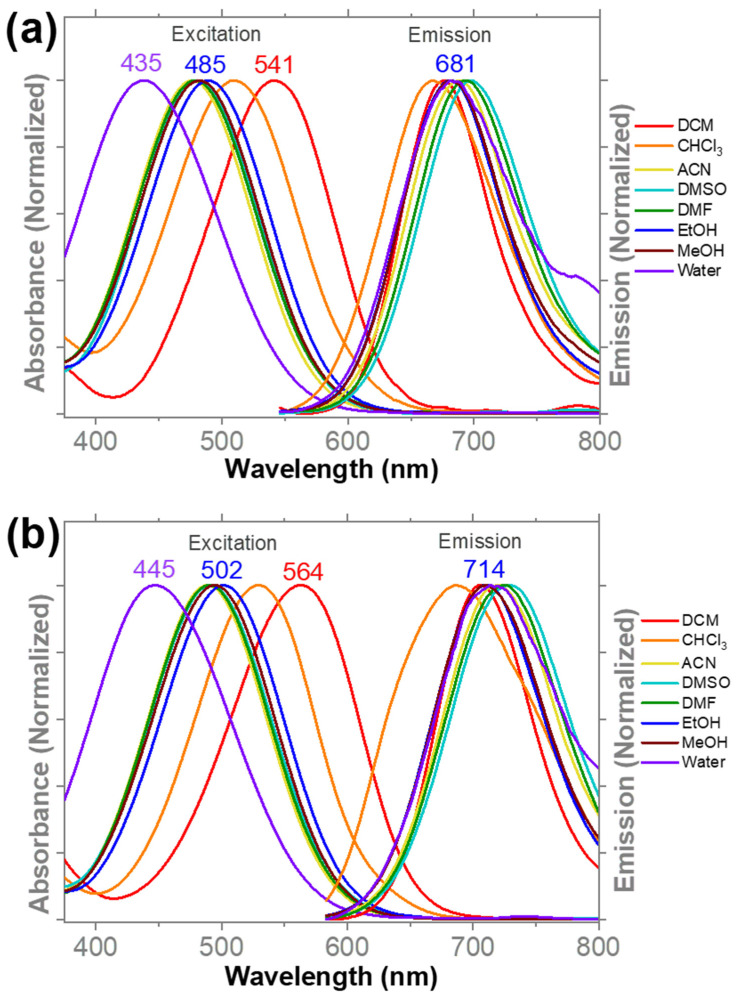
Normalized absorbance and emission spectra acquired for probe **1a** (**a**) and **1b** (**b**) in different solvents. Optical spectra were recorded for probes at 1 × 10^−6^ M and 25 °C. For the emission collection, probe **1a** was excited at 500 nm and **1b** was excited at 520 nm. Emission spectra were collected from 540 nm to 800 nm range.

Optical properties (absorbance, emission, fluorescence quantum yield, molar absorptivity and Stokes’ shift) of probes **1a** and **1b** were studied in different solvents and summarized in Table 1 and Figure 1. In summary, probes **1a** and **1b** exhibited excellent fluorescence quantum yields (ϕ_fl_ ≈ 0.24–0.72) and remarkably large Stokes’ shifts ∆λ ≈ 150–240 nm (Table 1). The absorbance and emission spectra of **1a** (λ_abs_ ≈ 530 nm; λ_em_ ≈ 690 nm) and **1b** (λ_abs_ ≈ 560 nm; λ_em_ ≈ 720 nm) exhibit a noticeable bathochromic shift (i.e., red-shift) in comparison to their predecessors 2-Di-1-ASP(λ_abs_ ≈ 465 nm; λ_em_ ≈ 590 nm) and 4-Di-1-ASP(λ_abs_ ≈ 475 nm; λ_em_ ≈ 605 nm), respectively (Scheme 1). This large bathochromic shift, observed in the optical spectra of **1** (∆λ_abs_ ≈ 80 nm; ∆λ_em_ ≈ 100 nm), is attributed to the extension of the π-spacer conjugation length (in between the donor-acceptor assembly) by adding an extra double bond (Scheme 1) [45]. Similar to 2-Di-1-ASP and 4-Di-1-ASP dyes, **1a** and **1b** also exhibited a similar trend in the shift in their optical spectra due to the “regio-effect” (see Scheme 1 and Figure 1). The para-methyl pyridinium linkage (i.e., **1b** and 4-Di-1-ASP) exhibited a noticeable bathochromic shift in their optical spectra (∆λ_abs_ ≈ 10–20 nm; ∆λ_em_ ≈ 20–40 nm), compared to the regio-isomeric ortho-methyl pyridinium linkage (i.e., **1a** and 2-Di-1-ASP), as a result of the extended donor-accepter interactions across the π-system via an additional resonance step (i.e., longer conjugation), as shown in the Scheme 1 (i.e., regio-effect). Probe **1** also exhibited higher fluorescence quantum yields (ϕ_fl_ ≈ 0.24–0.72) compared to the predecessors with shorter conjugation length (i.e., 2-Di-1-ASP and 4-Di-1-ASP) (Table 1). These results imply that the strength of the ICT interaction and length of the conjugated system play important roles towards the quantum efficiency of the fluorophores [46,47,48]. Both **1a** and **1b** were highly sensitive towards the nature of the solvent (i.e., polarity), thus changing solvent polarity from non-polar to polar showed a noticeable shift in the spectral position (Figure 1 and Table 1). In weakly polar solvents such as DCM or CHCl_3_, probe **1** exhibited a large bathochromic shift in the absorbance (λ_abs_ ≈ 540–560 nm) compared to polar protic solvents such as water or methanol (λ_abs_ ≈ 440–490 nm) (Figure 1). The observed negative solvatochromism (i.e., hypsochromic effect) in the absorption profile can be justified by considering the solvation-induced extra stabilization of the polar ground-state (i.e., HOMO level) compared to the less polar first-excited state of the probe **1** by polar protic solvents (i.e., water, ethanol or methanol) [49,50]. As a consequence, the energy gap in between HOMO-LUMO will rise, thus a noticeable hypsochromic shift (i.e., blue-shift) will occur in the absorption spectra [49,50]. Due to the positively charged nature of probe **1**, it is certain that polar solvents can significantly stabilize the probe’s ground-state than a non-polar or weakly polar solvent (i.e., ion-dipole interactions) [45]. Although it is not as prominent as in the absorption spectra, a careful observation will emphasize the solvatochromic nature of the emission properties of the probes (Figure 1 and Table 1). In weakly polar solvents (i.e., CHCl_3_), probe **1** exhibited an emission at λ_em_ ≈ 667–688 nm and in polar aprotic solvents, such as DMSO, a noticeable red-shift (i.e., bathochromic effect) was observed at λ_em_ ≈ 698–730 nm in the emission (Table 1 and Figure 1). As a result of the strong solvation interaction in polar solvent environments, a positive solvatochromic effect was thus observed for the probe’s emission spectra by lowering the energy of the emissive ICT state (i.e., strong ICT) [45,51]. Therefore, this strong optical response towards solvent polarity can be exploited as a sensing mechanism to develop robust environmentally sensitive fluorescent dyes. It is also important to note that moving from organic (i.e., DCM) to aqueous (i.e., water) environments leads to a significant decrease in the fluorescence quantum yields of the probes, which suggests their potential as “wash-free” stains for fluorescence microscopy imaging [7,11,52]. The observed significant quenching in fluorescence was reported as a solvent-assisted non-radiative pathway (i.e., quenching mechanism) that was studied extensively for solvatochromic organic fluorophores [53,54]. This observed low fluorescence quantum yield in aqueous environments is a well-known characteristic of many wash-free staining D-π-A type fluorescent imaging dyes [9]. 

To investigate the impact of the concentration towards optical properties, absorption and emission spectra were acquired at different probe concentrations from 10 µM to 50 µM (Figure S2). The absorption spectra of both **1a** and **1b** exhibited a linear regression, indicating no noticeable impact on the probe’s solubility within the tested concentration range (Figure S2). However, the emission spectra of both probes exhibited a moderate decrease in the increment, resulting in a non-linear regression at higher probe concentrations (i.e., 40 µM or higher). Since the absorbance spectra do not show this pattern, one can easily rule out the possibility of aggregation at higher concentrations. Therefore, this unusual trend in the emission intensities is likely to occur due to the inner filter effect at higher probe concentrations (Figure S2). As expected, probes exhibited a noticeable intensiometric response towards changes in viscosity of the solvent environment in a methanol:glycerol mixture exhibiting an excellent environmental sensitivity (Figure S3). Increasing solvent viscosity led to a significant improvement in the emission signals of **1a** and **1b**, where no changes in the absorption spectra were observed (Figure S3). Therefore, based on the experimental outcomes, these styryl pyridinium D-π-A systems will be very useful in developing viscosity-sensing NIR fluorescent dyes [55,56,57,58]. 

### 3.2. Sensing Human Serum Albumin (HSA) by Fluorometry

**Figure 2:**
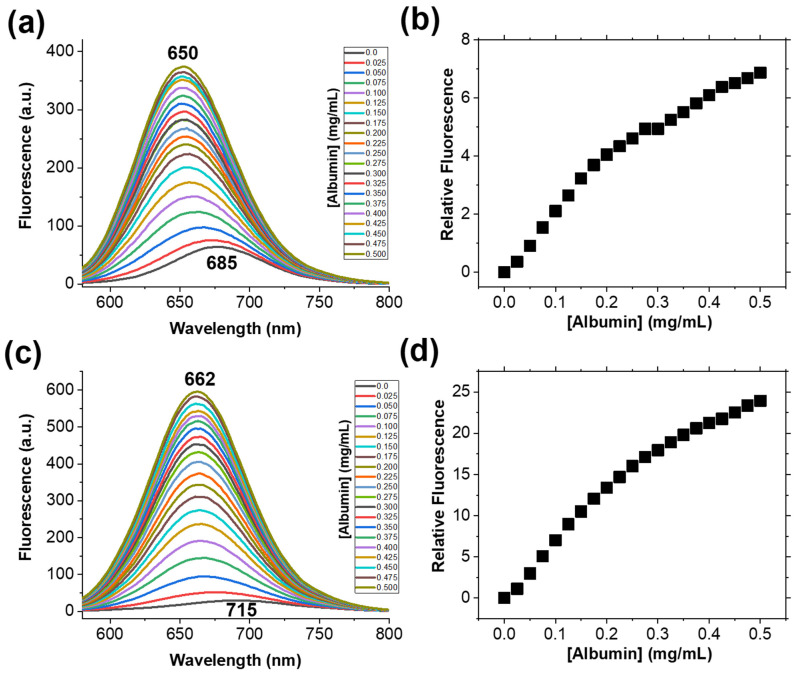
The fluorescence emission spectra acquired during the spectrometric titrations of probes **1a** (**a**) and **1b** (**c**) at 1 × 10^−6^ M concentration with a 5% HSA solution in water at room temperature. Figures (**b**) and (**d**) represents the relative fluorescence signal recorded at the emission maxima as a function of HSA concentration in the solution for probes **1a** and **1b**, respectively. Probes were excited at 520 nm and the emission spectra were collected from 540 nm to 800 nm range.

Fluorescence quantum yields calculated for the probes exhibited a significant decrease while moving from organic (non-polar) solvents to aqueous (polar) solvents (Table 1). With this observation, we hypothesized that probe **1** will produce a large intensiometric response in emission if successfully internalized in to a hydrophobic environment from the aqueous solution. To test this hypothesis, we have introduced human serum albumin (HSA), a well-known water-soluble small molecule binding transporter protein in to the aqueous environment [39,59,60]. Spectrometric titrations were performed for probes **1a** and **1b** in the aqueous solutions with 5% HSA in room-temperature (Figure 2). As we hypothesized, probe **1a** and **1b** exhibited a significant fluorescence enhancement (by 7 fold and 24 fold, respectively) upon addition of HSA, indicating possible internalization in to the hydrophobic binding pockets in HSA of the protein (Figure 2) [39,61]. It is important to note that probe **1b** exhibited a higher sensitivity (i.e., 3 times than **1a**) towards HSA than **1a** based on experimental findings (Figure 2). This high sensitivity observed for **1b** indicates distinct advantages of its structural geometry (see Scheme 1) towards efficient binding interactions (i.e., hydrophobic interactions) [39]. 

The limit of detection (LOD) and limit of quantification (LOQ) values were calculated for both probes by extracting linearly fitted regions of the probe’s response curves via regression analysis. The calculated LOD values for probes **1a** and **1b** were 0.059 mg/mL and 0.043 mg/mL, respectively (Table S1). During the spectrometric titrations, the absorption spectra of the probes did not exhibit any noticeable changes, which is indicative of no structural changes or covalent interactions taking place upon binding events (Figure S4). Besides this large intensiometric response in fluorescence signal, a highly noticeable blue-shift (i.e., hypsochromic effect) in the emission profiles was also observed for **1a** (λ_em_ ≈ 685 nm to 650 nm) and **1b** (λ_em_ ≈ 715 nm to 662 nm) during the spectrometric titrations (Figure 2). This large hypsochromic effect is indicative of possible geometric stabilization of the probe in hydrophobic protein environments to enhance binding interactions, which was discussed during previous work [39,62,63]. The probe-albumin binding events were validated by measuring intrinsic fluorescence quenching of HSA at λ_em_ ≈ 346 due to relocation of the protein towards more hydrophobic environments via hydrophobic probe-protein interactions (ESI Figure S4) [64,65,66,67]. In addition, the solution stability of probe-albumin complexes were analyzed in aqueous solution by recording optical spectra (absorbance and emission) of the probe–protein complex as a function of time (Figure S5). Probe **1a** and **1b**’s albumin complexes exhibited stable fluorescence signals over 2 h, indicating an excellent stability in the solution (Figure S5). Therefore, the architecture of probe **1** will be highly useful in developing potential albumin sensing fluorescent dyes that can be used in aqueous biological environments. Since **1a** and **1b** are positively charged, they were tested against various anionic species (~10 equivalence) in solution to determine any possible interactions (i.e., electrostatic). Based on the optical spectra analysis, the probes did not exhibit any noticeable response towards any of the anionic species tested (Figure S6).

### 3.3. Low-Temperature and Fluorescence Lifetime Studies

**Figure 3:**
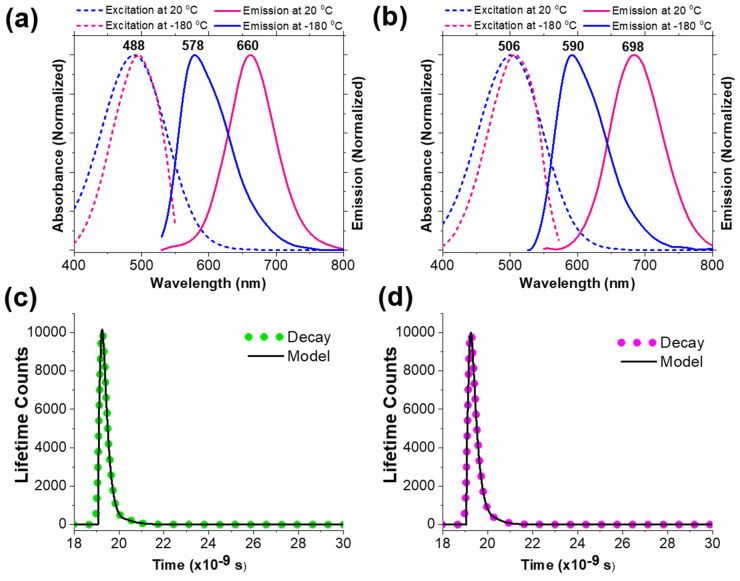
The low-temperature excitation and emission analysis for probes **1a** (1 × 10^−6^ M; (**a**)) and **1b** (1 × 10^−6^ M; (**b**)) in absolute ethanol. **1a** and **1b** were excited at 485 nm and 505 nm, respectively. (**c**,**d**) represents data obtained by using the time-correlated single-photon counting (TCSPC) method for lifetime measurements. Sample: solutions of dye **1a** (**c**) and **1b** (**d**) in acetonitrile (under Ar). Excitation: 485 nm Lasers. Data were collected by using a 650 nm long path filter. Reference: scattering solution. Result: τ1 = 0.20 ns, τ2 = 0.80 ns; χ^2^ = 1.12 for **1a** and τ1 = 0.23 ns, τ2 = 0.44 ns; χ^2^ = 0.96 for **1b**.

In order to understand the significance of ICT and the impact of the regio-effect in two isomeric probe designs (i.e., **1a** and **1b**), we acquired low temperature fluorescence spectra for the probes in ethanol (Figure 3). Ethanolic solutions of **1a** and **1b** were frozen to −180 °C in a liquid nitrogen dewer instantly to limit molecular rotations and bond reorganizations that are associated with the subsequent ICT process. At ultra-low temperatures (i.e., −180 °C), **1a** and **1b** exhibited their emissions at λ_em_ ≈ 578 nm and λ_em_ ≈ 590 nm, respectively (Figure 3a,b) The significant blue-shift observed in the emission spectra suggested that, at a frozen matrix (i.e., −189 °C), the ICT process is hindered due to restricted molecular rotation. Thus, a large hypsochromic shift in the emission spectra (∆λ ≈ 82 nm for **1a** and ∆λ ≈ 108 nm for **1b**) was observed. Most importantly, the acquired excitation spectra did not change at low-temperature conditions (i.e., −180 °C), suggesting no structural changes associated with the frozen matrix conditions (Figure 3a,b). These data clearly indicate that the observed NIR emission in probe **1** solely depends on the efficiency of the ICT process. The fluorescence lifetime measurement of **1a** revealed a two-exponential decay pattern with τ1 = 0.20 ns (95.8%) and τ2 = 0.80 ns (4.2%) with the best curve fitting of reduce chi-square, χ^2^_r_ = 1.12 (Figure 3c) in acetonitrile. Similarly, **1b** exhibited a two-exponential decay with τ1 = 0.23 ns (91.7%) and τ2 = 0.43 ns (8.3%) with the best curve fitting of reduce chi-square, χ^2^_r_ = 0.96 (Figure 3d). The two observed exponential decay patterns with two lifetime components can be attributed to the two excited states (i.e., LE and ICT), as reported in previous findings for similar probes [68].

### 3.4. Visualizing Microorganisms via Fluorescence Microscopy

**Figure 4:**
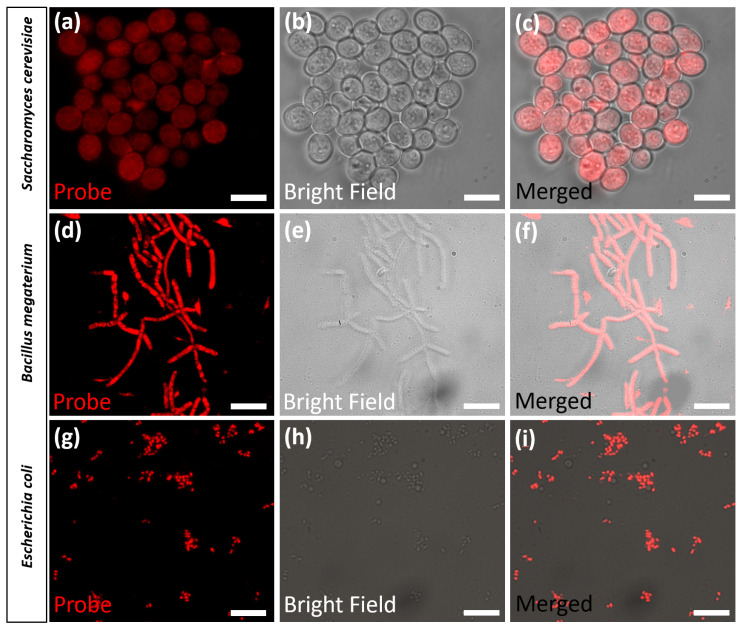
Fluorescence microscopy images obtained from *Saccharomyces cerevisiae* (**a**–**c**), *Bacillus megaterium* (**d**–**f**)*,* and *Escherichia coli* (**g**–**i**), stained with probe **1a** (10 µM) for 30 min. Images (**a**–**i**) represents, probe **1a** emission in the cells (**a**,**d**,**g**), bright field images (**b**,**e**,**h**), and merged images (**c**,**f**,**i**). Images were acquired by exciting the stained organisms with 532 nm laser line with standard Cy3 filter settings (580–620 nm) for the emission collection. The scale bar indicates a 20-micron region.

**Figure 5:**
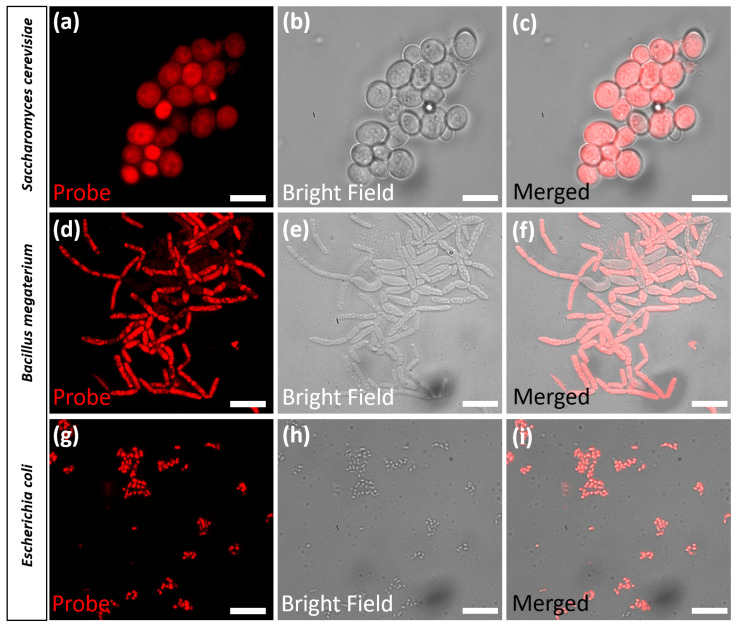
Fluorescence microscopy images obtained from *Saccharomyces cerevisiae* (**a**–**c**), *Bacillus megaterium* (**d**–**f**)*,* and *Escherichia coli* (**g**–**i**), stained with probe **1b** (10 µM) for 30 min. Images (**a**–**i**) represents, probe **1b** emission in the cells (**a**,**d**,**g**), bright field images (**b**,**e**,**h**), and merged images (**c**,**f**,**i**). Images were acquired by exciting the stained organism with 532 nm laser line with standard Cy3 filter settings (580–620 nm) for the emission collection. The scale bar indicates a 20-micron region.

Some recent research have highlighted the potential of styryl pyridinium dyes as excellent fluorescent bacterial stains (i.e., *Escherichia coli*) for fluorescent microscopy applications [14,69]. Also, several commercially available fluorescent bacterial stains, such as FM-1-43, consist of styryl pyridinium moiety in the structure [4]. Therefore, we hypothesized that probe **1a** and **1b** will be ideal candidates for staining bacteria for fluorescence microscopy imaging. Since probe **1** exhibited outstanding photophysical properties (i.e., bright NIR emission, high fluorescence quantum yield, longer wavelength excitation and large Stokes’ shift) that are suitable for a potential imaging candidate, we decided to perform fluorescence microscopy imaging experiments with different microorganisms (*Bacillus megaterium*, *Escherichia coli*, and *Saccharomyces cerevisiae*) [9]. Thus, two bacterial cultures, *Bacillus megaterium* (Gram-positive) and *Escherichia coli* (Gram-negative), were grown in appropriate growth media and stained with varying concentrations of probe **1a** and **1b** (5 µM, 10 µM and 20 µM), as explained in the methodology. Interestingly, both *Bacillus megaterium* and *Escherichia coli* cells stained with probe **1a** and **1b** exhibited bright emission patterns indicating the probe’s potential to visualize both Gram-positive and Gram-negative bacterial strains (Figure 4 and Figure 5) by fluorescence microscopy. The uniform bright-red emission patterns arising from the stained bacterial cells indicated that the probes are likely visualizing the plasma membranes of the strains, as reported in previous findings [14,69]. Also, it is important to note that probes (**1a** and **1b**) did not produce background interference/noise during fluorescence microscopy imaging experiments. This characteristic of the probe can be attributed to the significant fluorescent quantum yield (or brightness) difference observed from organic (i.e., DCM) vs. aqueous (i.e., water) environments, which will likely facilitate bright fluorescence emission upon internalization into hydrophobic cellular environments of the microorganisms such as the plasma membrane (Table 1). Therefore, these probes (**1a** and **1b**) will be ideal candidates for wash-free fluorescence imaging applications where the post-staining washing step is impossible to perform without perturbation to the specimen. Previously reported work showed that small-molecule cationic probes, such as **1**, may likely interact with the surface of the microorganism via electrostatic and/or hydrophobic interactions [70,71]. Therefore, it is certain that the positively charged nature of the probe plays a key role towards observed specificity [70,71,72,73]. In order to further evaluate the potential of these probes to visualize other microorganisms, we decided to stain yeast cells (*Saccharomyces cerevisiae*) with 10 µM concentration of the probe (**1a** or **1b**) for fluorescence microscopy imaging (Figure 4 and Figure 5). Interestingly, yeast cells stained with probe **1** exhibited uniform bright-red fluorescence patterns similar to what was observed for bacterial species, thus indicating possible internalization in to the cells (Figure 4 and Figure 5). The observed bright red fluorescence also suggested that probes may likely accumulate in to the hydrophobic cores within the cells (i.e., plasma membrane and/or organelle membranes) and not in to the aqueous cytoplasm. However, unfortunately, no further studies were possible due to the limited availability of the imaging infrastructure.

## 4. Conclusions

In summary, two styryl-pyridinium containing fluorescent imaging dyes (**1a**–**1b**) were developed by utilizing donor-π-acceptor (D-π-A) architecture in the presence of strong donor groups (-NMe_2_). Due to the presence of a strong electron donating group (i.e., -NMe_2_), probes exhibited strong intramolecular charge transfer (ICT) activity, which evolved to very large Stokes’ shifts (∆λ > 150 nm). Probes exhibited bright near-infrared fluorescence (λ_em_ ≈ 690–730 nm; ϕ_fl_ ≈ 0.24–0.72) and high-environment sensitivity (i.e., solvent polarity, viscosity, and temperature). Probe **1b** exhibited a noticeable bathochromic shift in optical spectra when compared to its regio-isomer **1a** due to the regio-effect, which enables extended ICT interaction across the conjugated system via resonance. The impact of the ICT process was studied by low-temperature fluorescence analysis. Probes (**1a** and **1b**) exhibited an excellent ability to quantify human serum albumin in aqueous solutions by fluorometry with calculated LOD values at 0.059 mg/mL (**1a**) and 0.043 mg/mL (**1b**), respectively. The NIR emission, extremely large fluorescence quantum yield difference in organic vs aqueous environments and longer wavelength excitation profile, suggested that probes **1a** and **1b** are ideal fluorescent staining agents for bioimaging applications. Probes exhibited exceptional potential to visualize Gram-positive and Gram-negative bacteria (i.e., *Bacillus megaterium*, *Escherichia coli*) and yeast (i.e., *Saccharomyces cerevisiae*) by fluorescence microscopy with no background interference. Based on these highly desirable and unique photophysical properties with its microorganism staining ability, probe **1** will provide powerful molecular designs in the near future to develop robust fluorescent imaging dyes.

## Figures and Tables

**Table 1:** Spectroscopic properties of probes **1a** and **1b**.

Solvent	DCM	CHCl_3_	ACN	DMSO	DMF	EtOH	MeOH	Water
**Probe 1a**								
**λ_abs_ (nm)**	541	512	478	477	476	488	482	438
**λ_em_ (nm)**	678	667	687	698	694	684	688	687
**∆λ (nm)**	137	155	209	221	218	196	206	249
**ϕ_fl_**	0.61	0.29	0.33	0.68	0.40	0.24	0.38	0.01
**ε** **(M^−1^·cm^−1^)**	47,023	42,456	41,482	41,470	40,700	42,428	39,647	33,162
**Probe 1b**								
**λ_abs_ (nm)**	561	530	487	489	490	501	492	446
**λ_em_ (nm)**	706	688	718	731	724	714	712	718
**∆λ (nm)**	145	158	231	242	234	213	220	272
**ϕ_fl_**	0.72	0.47	0.24	0.61	0.48	0.38	0.31	0.005
**ε** **(M^−1^·cm^−1^)**	48,120	43,442	43,100	49,406	42,100	42,159	40,477	25,670

## Data Availability

Not applicable.

## Supplementary Materials

The following supporting information can be downloaded at: https://www.mdpi.com/article/10.3390/bios13080799/s1, Figure S1: Characterization data; Figure S2: Concentration dependency of the optical properties; Figure S3: Viscosity dependency of the optical properties; Figure S4: Spectrometric titration of the probes with HSA; Figure S5: Stability evaluation for the probe-albumin complex; Figure S6: Sensitivity of the probes towards anions and other species; Figure S7: Solid-state emission spectra analysis; Table S1: Limit of detection (LOD) and limit of quantification (LOQ) calculation.
