# Supplementary material for: Bright NIR-Emitting Styryl Pyridinium Dyes with Large Stokes’ Shift for Sensing Applications
Source: Biosensors (Basel). 2023 Aug 9;13(8):799. doi: 10.3390/bios13080799 (PMC10452306; doi:10.3390/bios13080799)
Supplement: Supplementary file 1 [file biosensors-13-00799-s001.zip › biosensors-2523623-supplementary.pdf]

Bright NIR-Emitting Styryl Pyridinium Dyes with Large Stokes’ Shift for Sensing Applications

Supporting Information

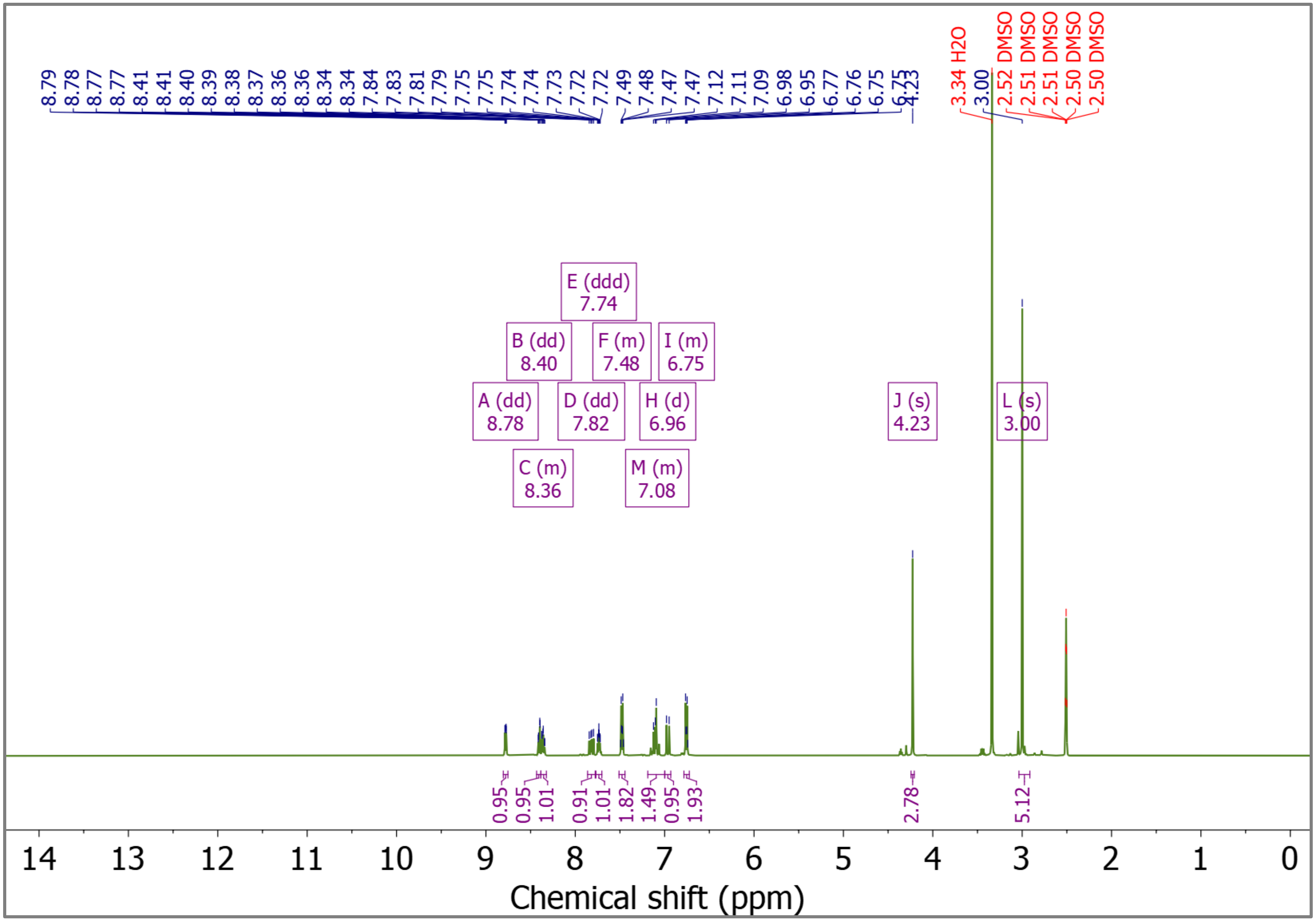

Figure S1.1 ^{1}H NMR spectra of **1a** (500 MHz in DMSO-*d6*).

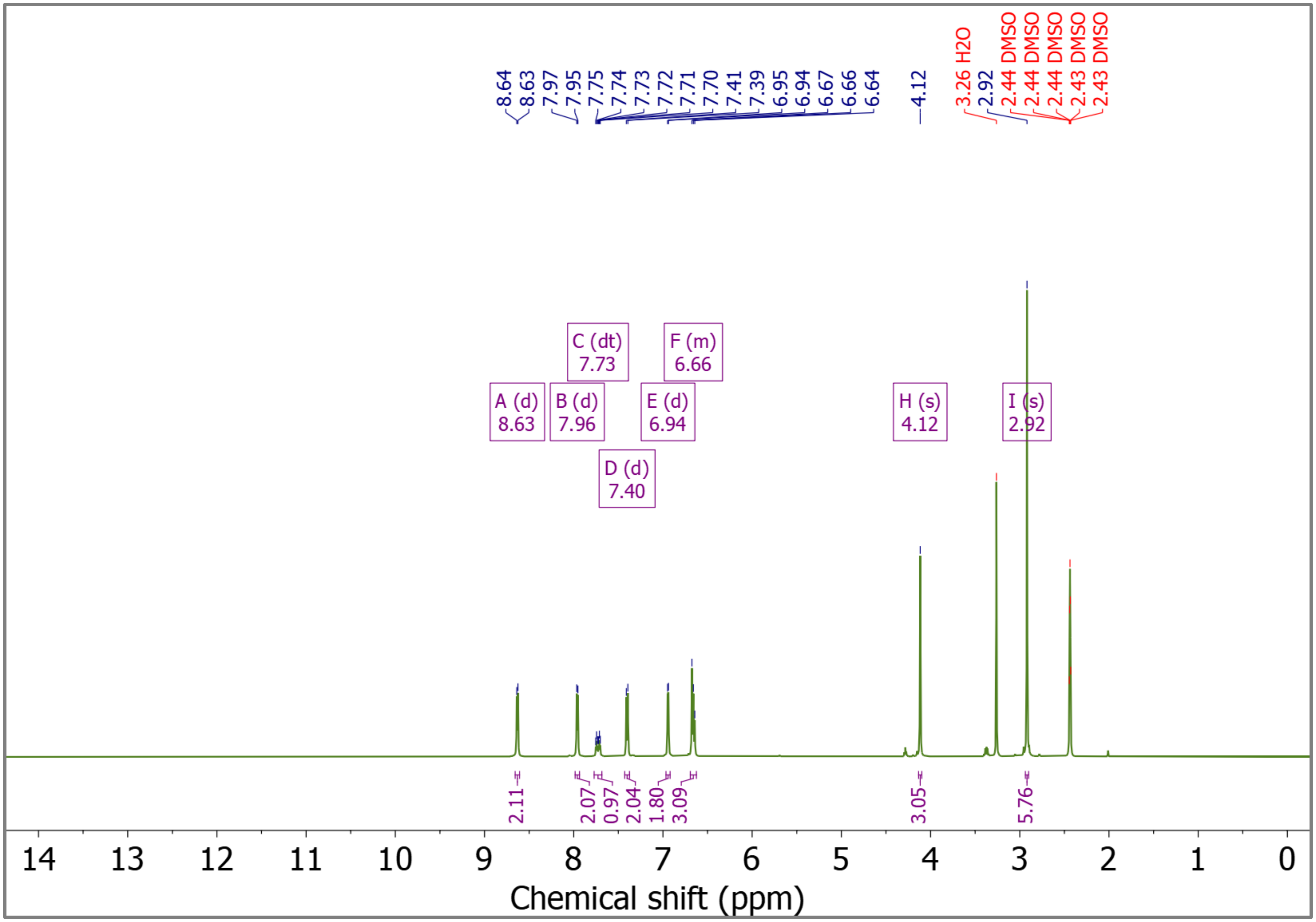

Figure S1.2 ^{1}H NMR spectra of **1b** (500 MHz in DMSO-*d6*).

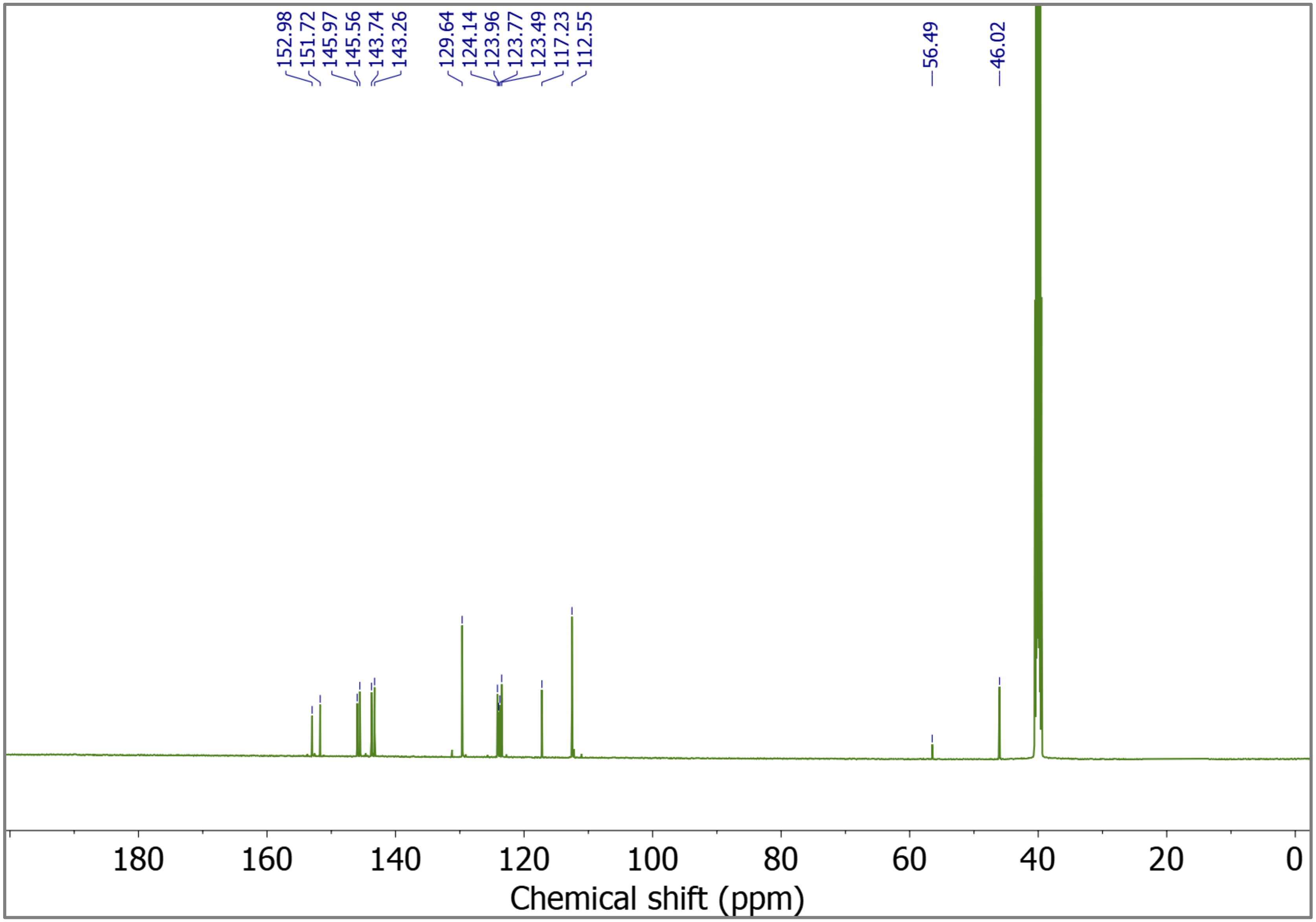

Figure S1.3 ^{13}C NMR spectra of **1a** (126 MHz in DMSO-*d6*).

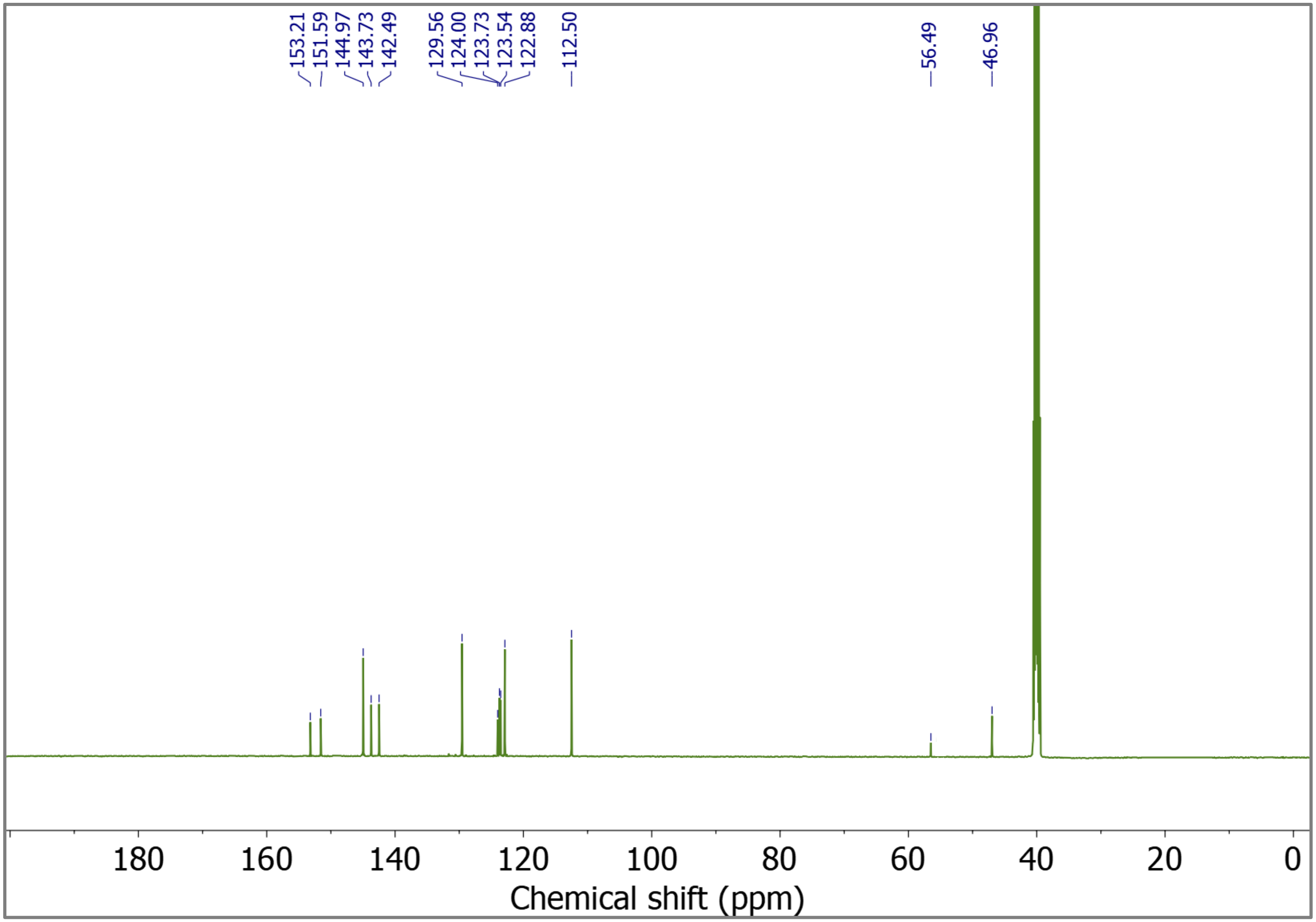

Figure S1.4 ^{13}C NMR spectra of **1b** (126 MHz in DMSO-*d6*).

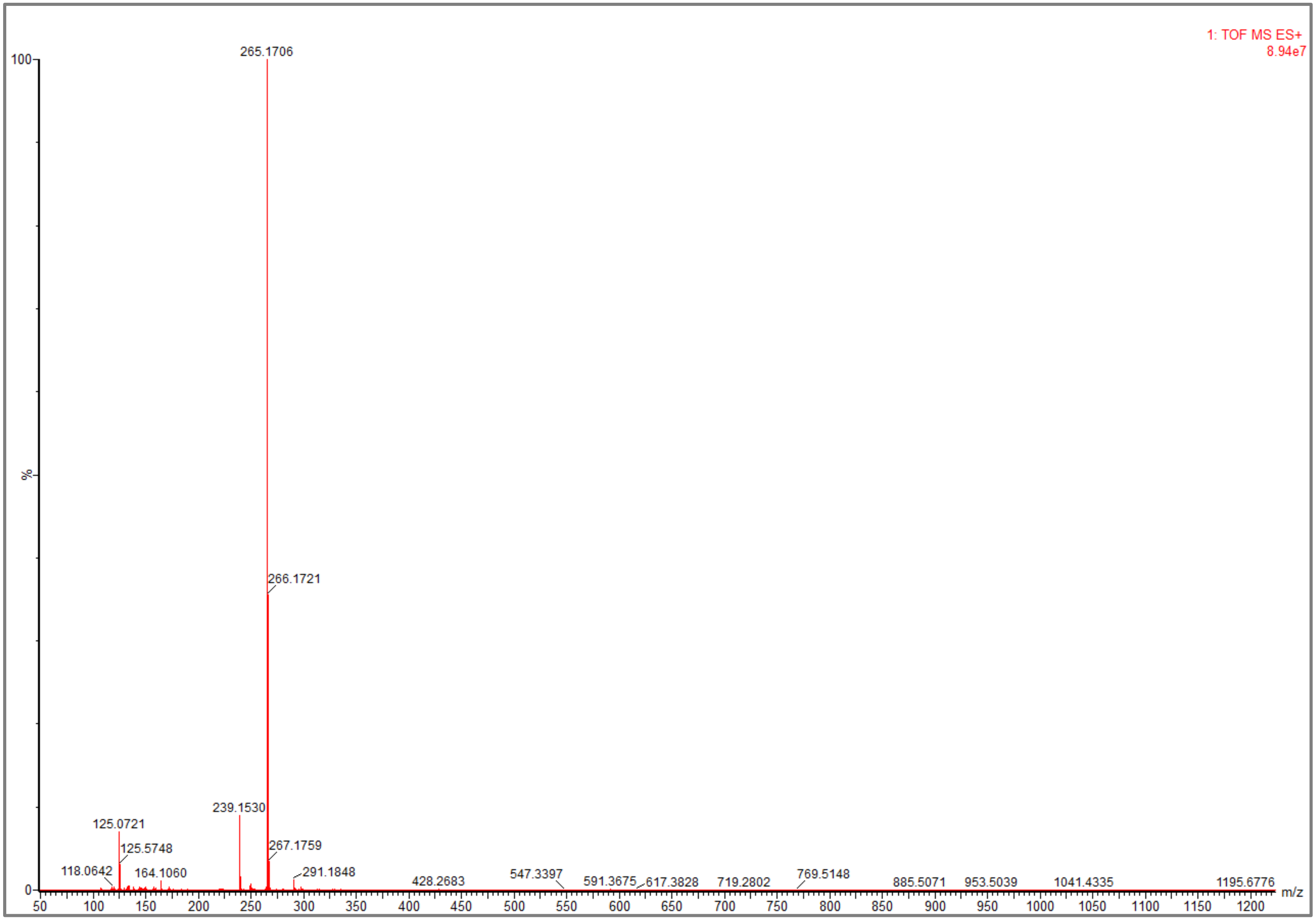

Figure S1.5 High-resolution mass spectra (TOF MS ES+) of **1a.**

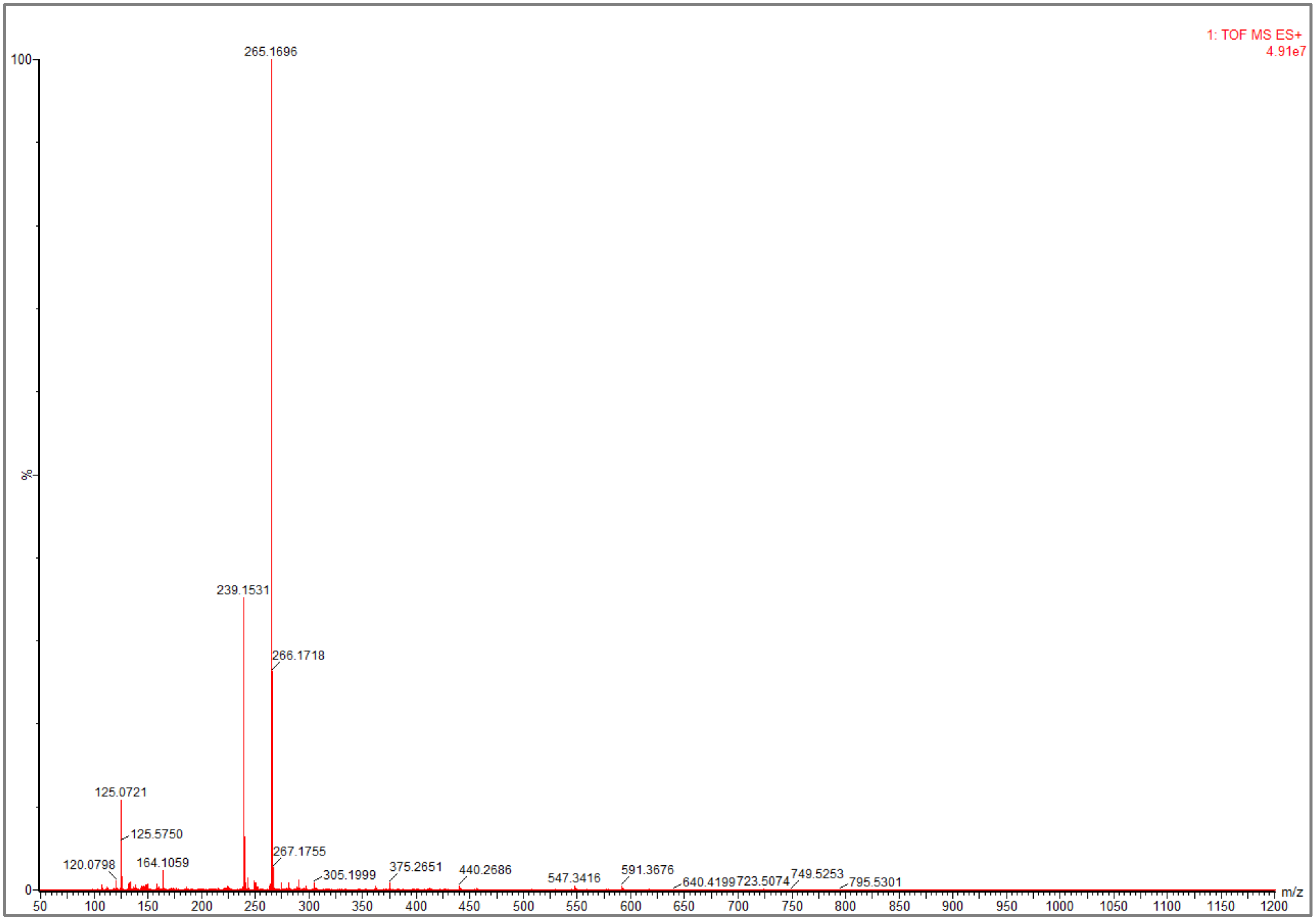

Figure S1.6 High-resolution mass spectra (TOF MS ES+) of **1b.**

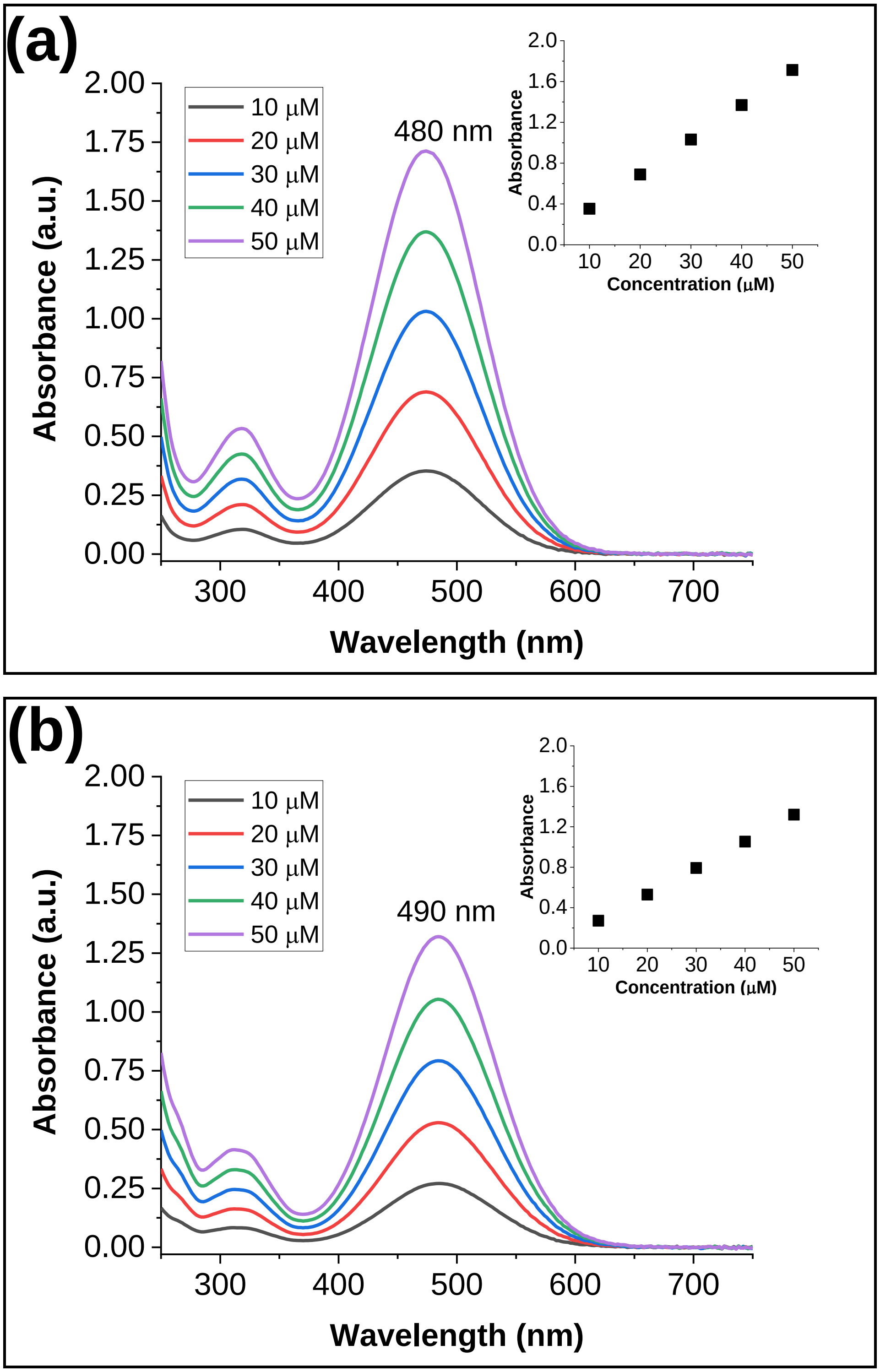

Figure S2.1 Absorbance spectra acquired for probe **1a** (a) and **1b** (b) under different concentrations at room temperature.

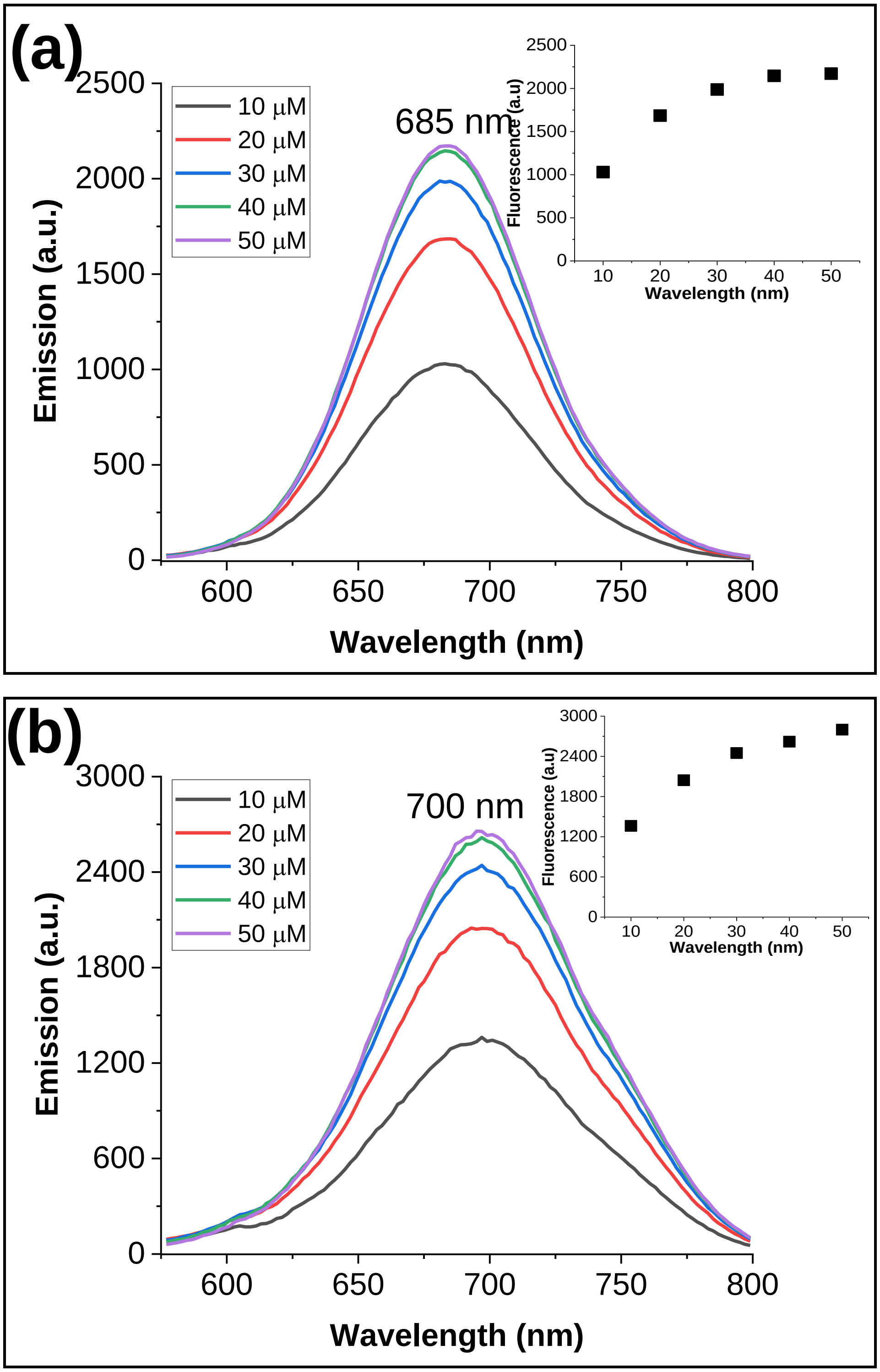

Figure S2.2 Emission spectra acquired for probe **1a** (a) and **1b** (b) under different concentrations at room temperature. Probes were excited at 500 nm and the emissions were collected from 530nm to 800 nm.

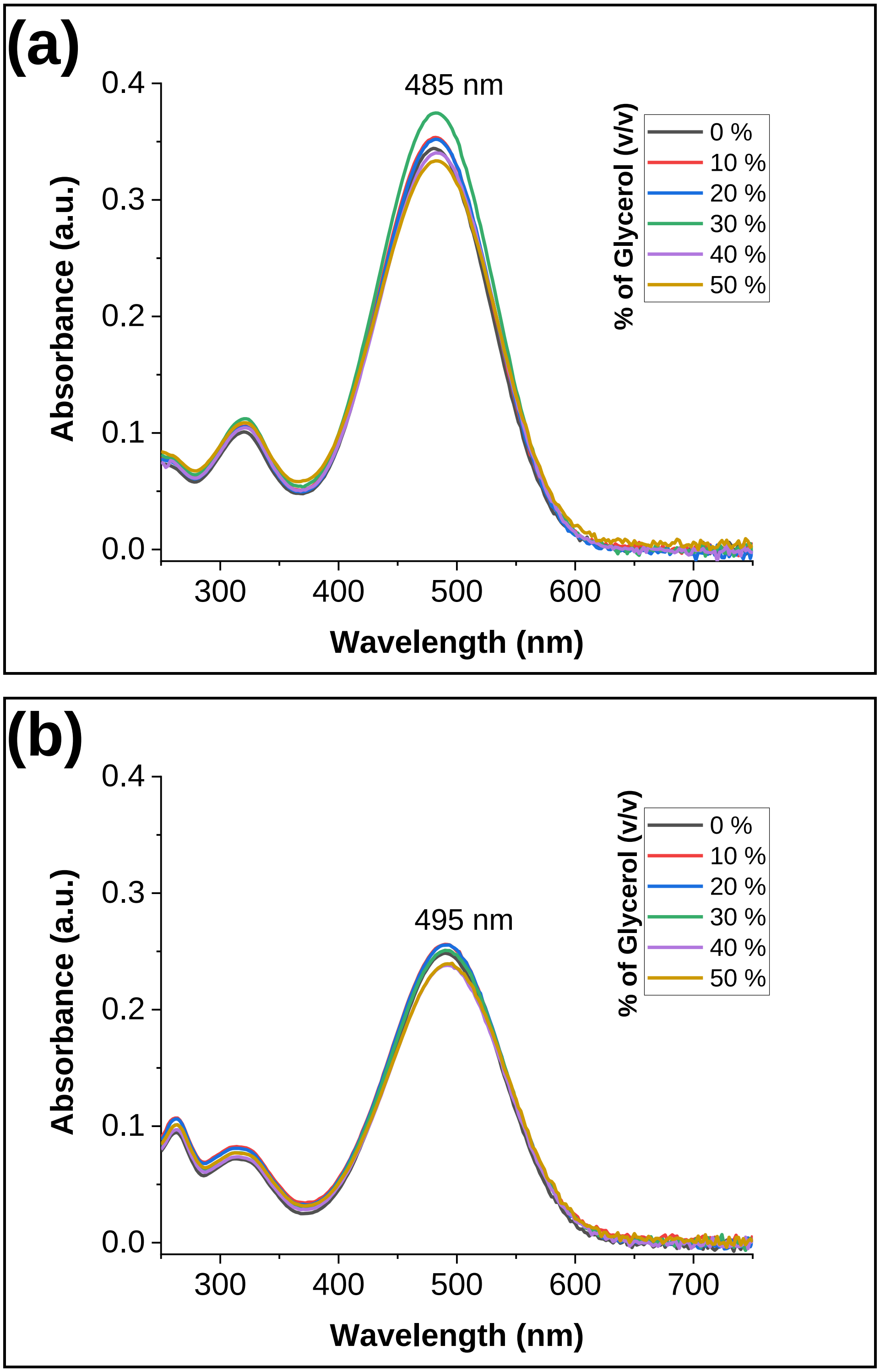

Figure S3.1 Absorbance spectra acquired for probe **1a** (a) and **1b** (b) in different Glycerol:MeOH % at room temperature.

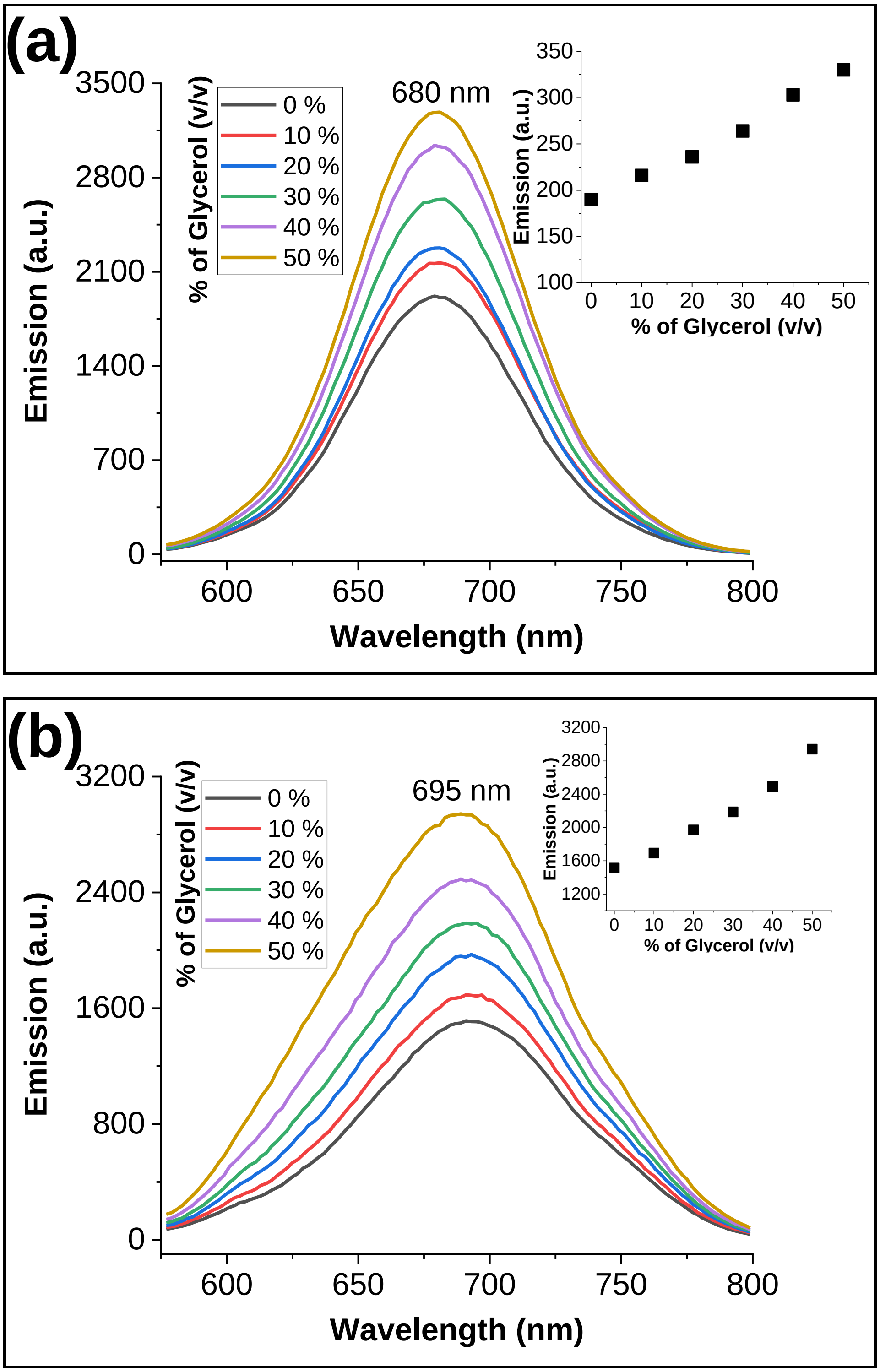

Figure S3.2 Emission spectra acquired for probe **1a** (a) and **1b** (b) in different Glycerol:MeOH % at room temperature. Probes were excited at 500 nm and the emissions were collected from 530nm to 800 nm.

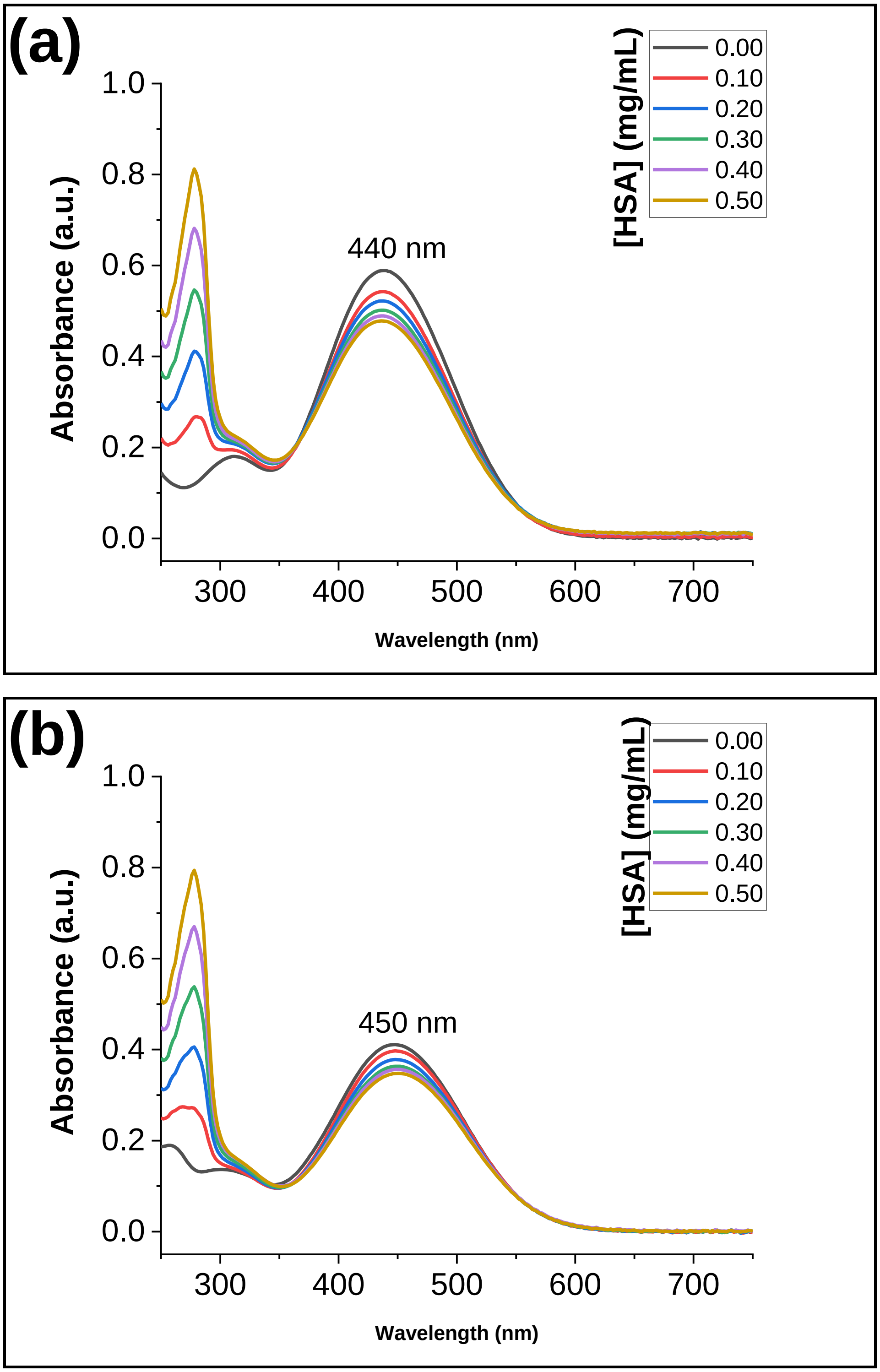

Figure S4.1 Absorbance spectra acquired for probe **1a** (a) and **1b** (b) (10×10^{-6} M) with the spectrometric titration with 5 % HSA in water at room temperature.

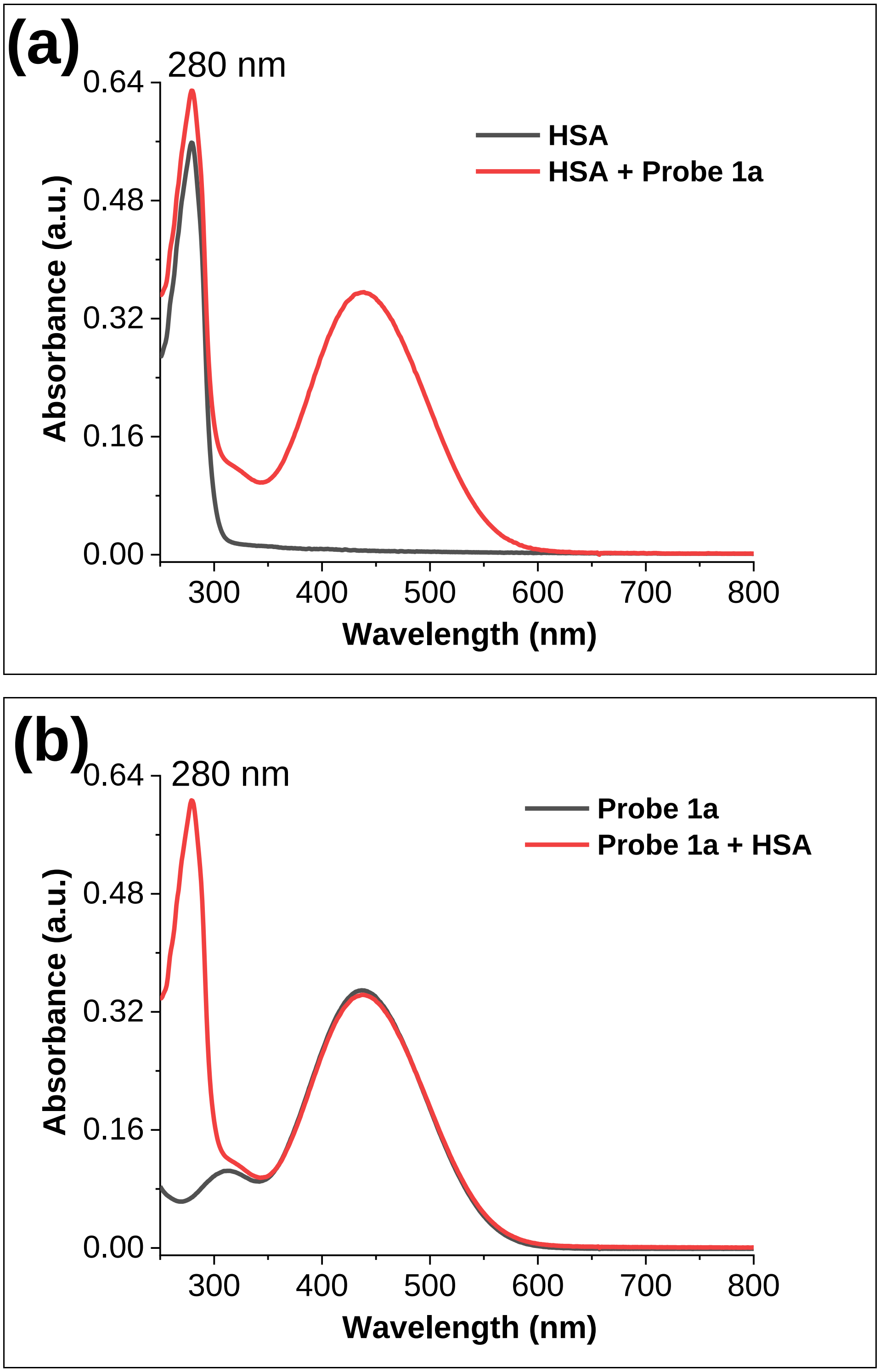

Figure S4.2 Absorbance spectra acquired for probe **1a** (10 x 10^{-6} M) with the different addition sequences of HSA (1.1 equivalent) in water at room temperature.

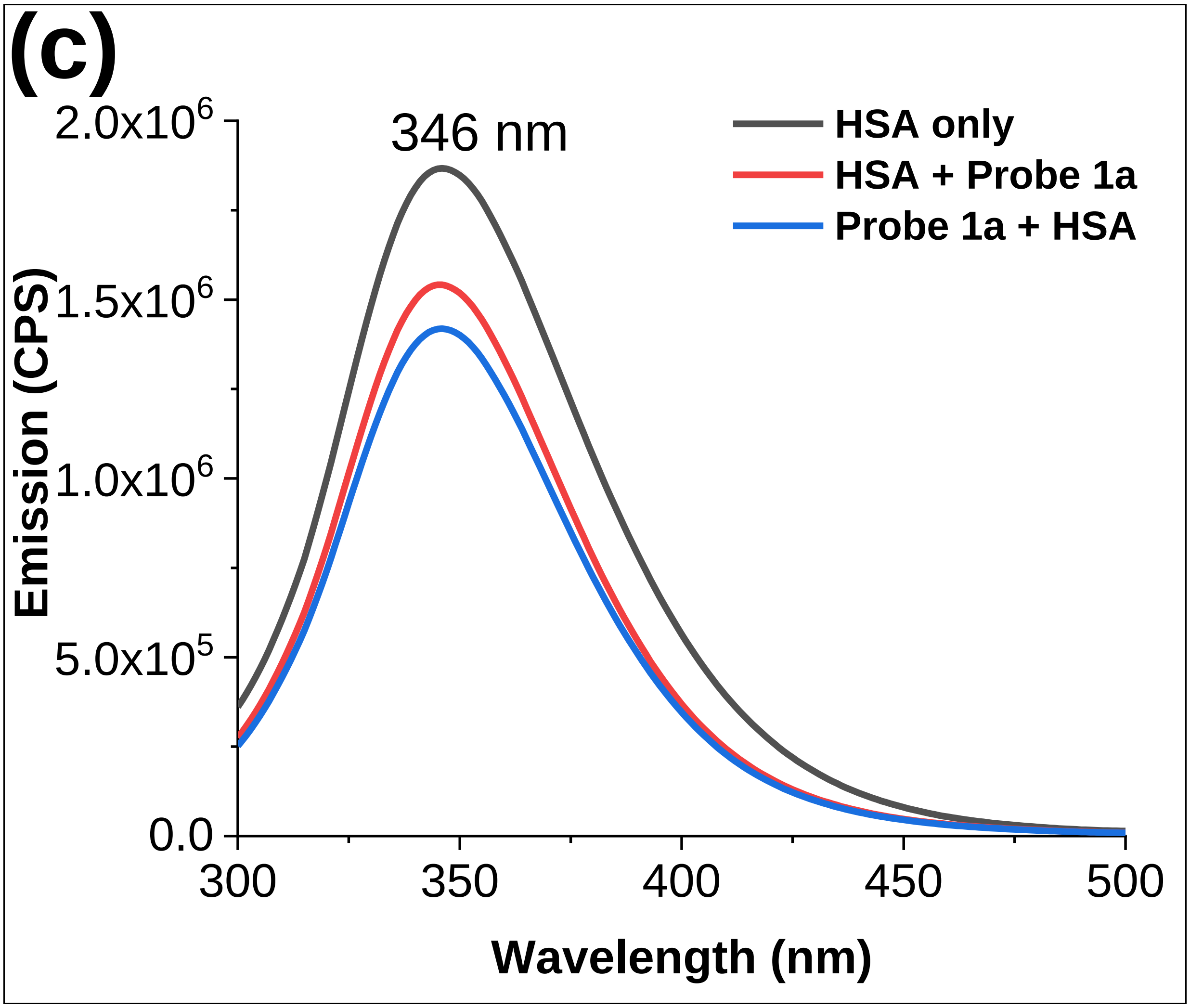

Figure S4.3 The intrinsic fluorescence spectra acquired for HSA (1.1 x 10^{-5} M) with different addition sequences of probe **1a**. The spectra represent the emission of HSA only (black), emission of HSA+**1a** (red) and emission of **1a**+HSA. The emission spectra were recorded by exciting the solution at 280 nm while collecting emission from 295 nm to 500 nm.

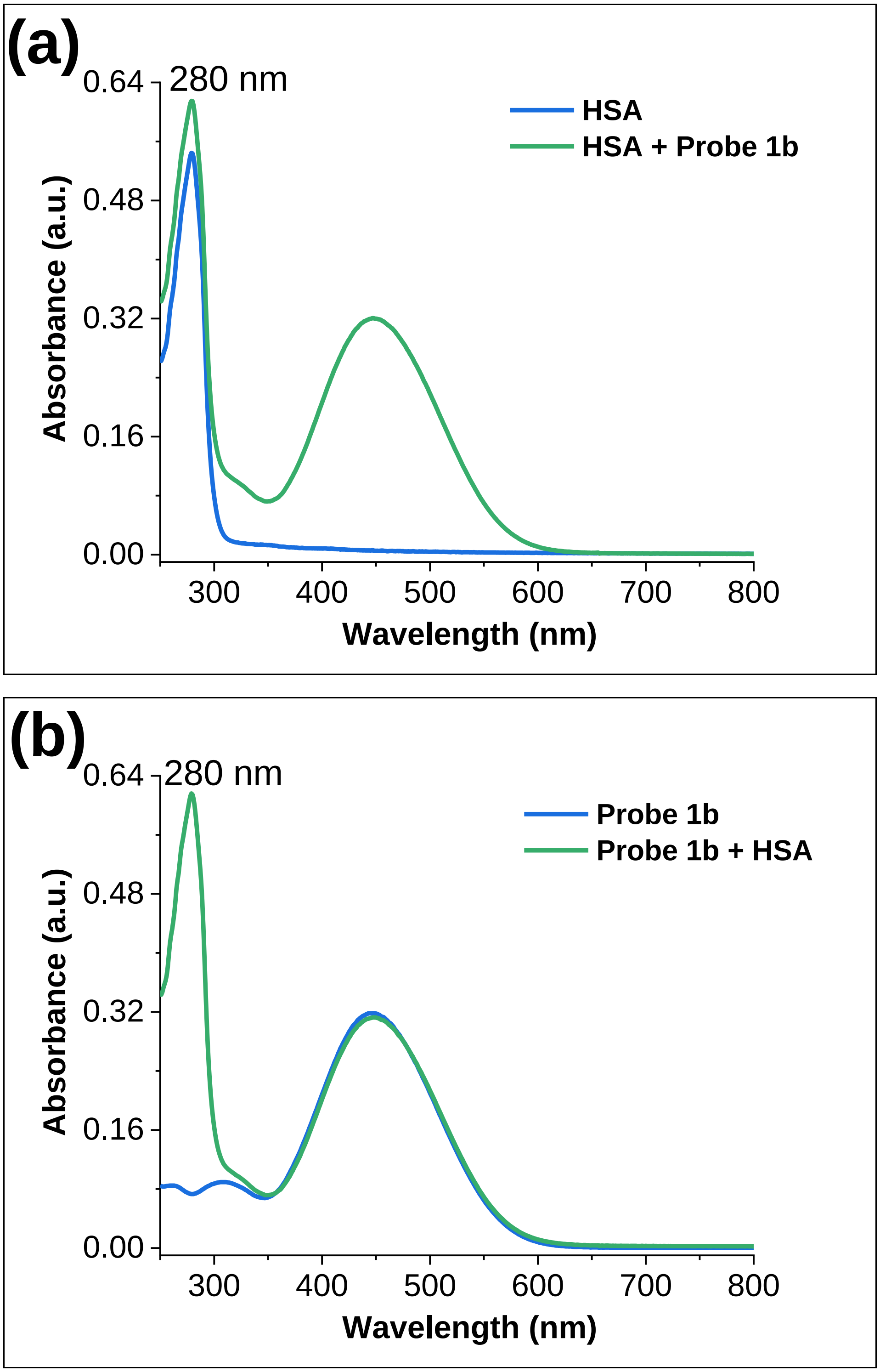

Figure S4.4 Absorbance spectra acquired for probe **1b** (10×10^{-6} M) with the different addition sequences of HSA (1.1 equivalent) in water at room temperature.

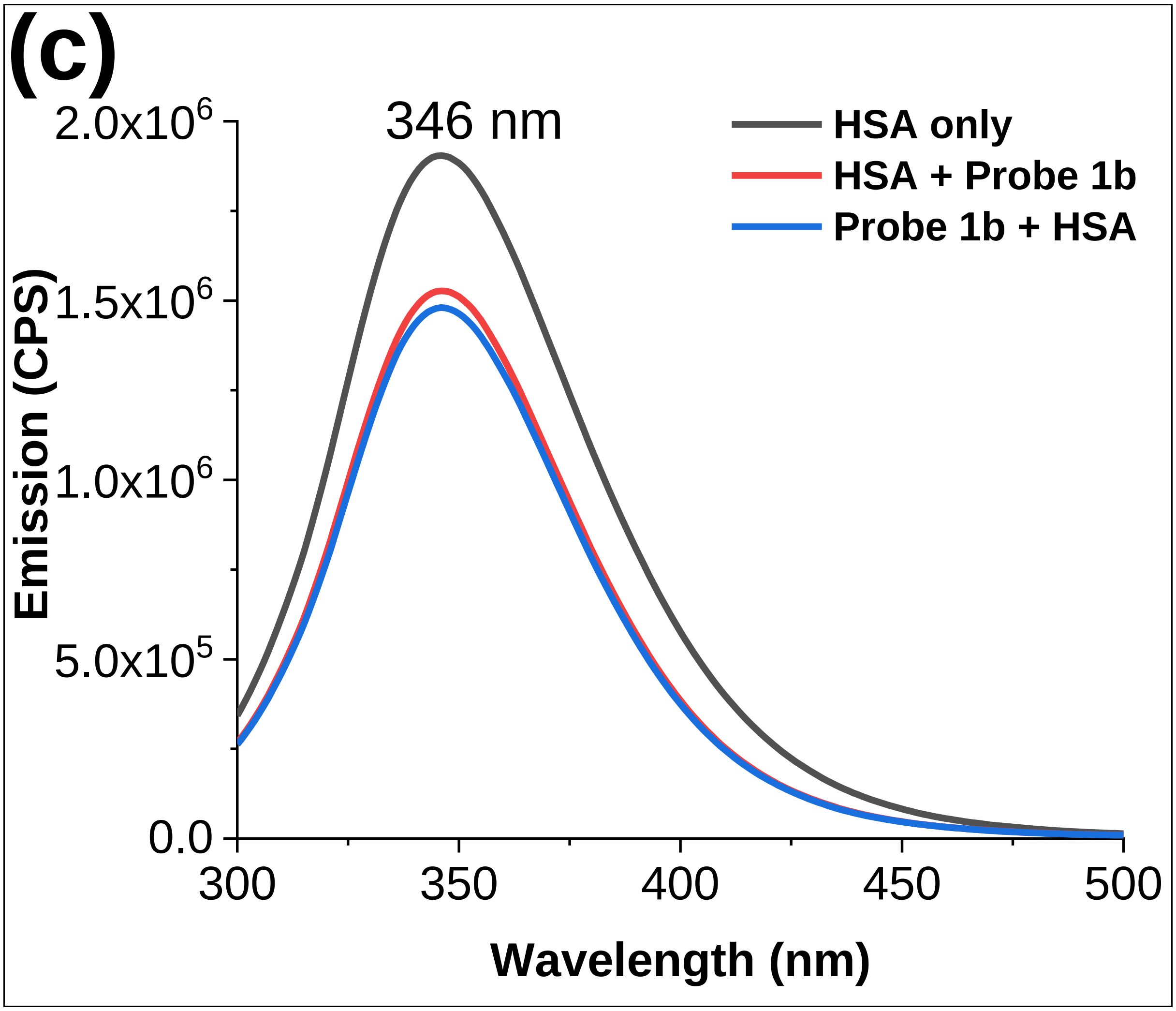

Figure S4.5 The intrinsic fluorescence spectra acquired for HSA (1.1 x 10^{-5} M) with different addition sequences of probe **1b**. The spectra represent the emission of HSA only (black), emission of HSA+**1b** (red) and emission of **1b**+HSA. The emission spectra were recorded by exciting the solution at 280 nm while collecting emission from 295 nm to 500 nm.

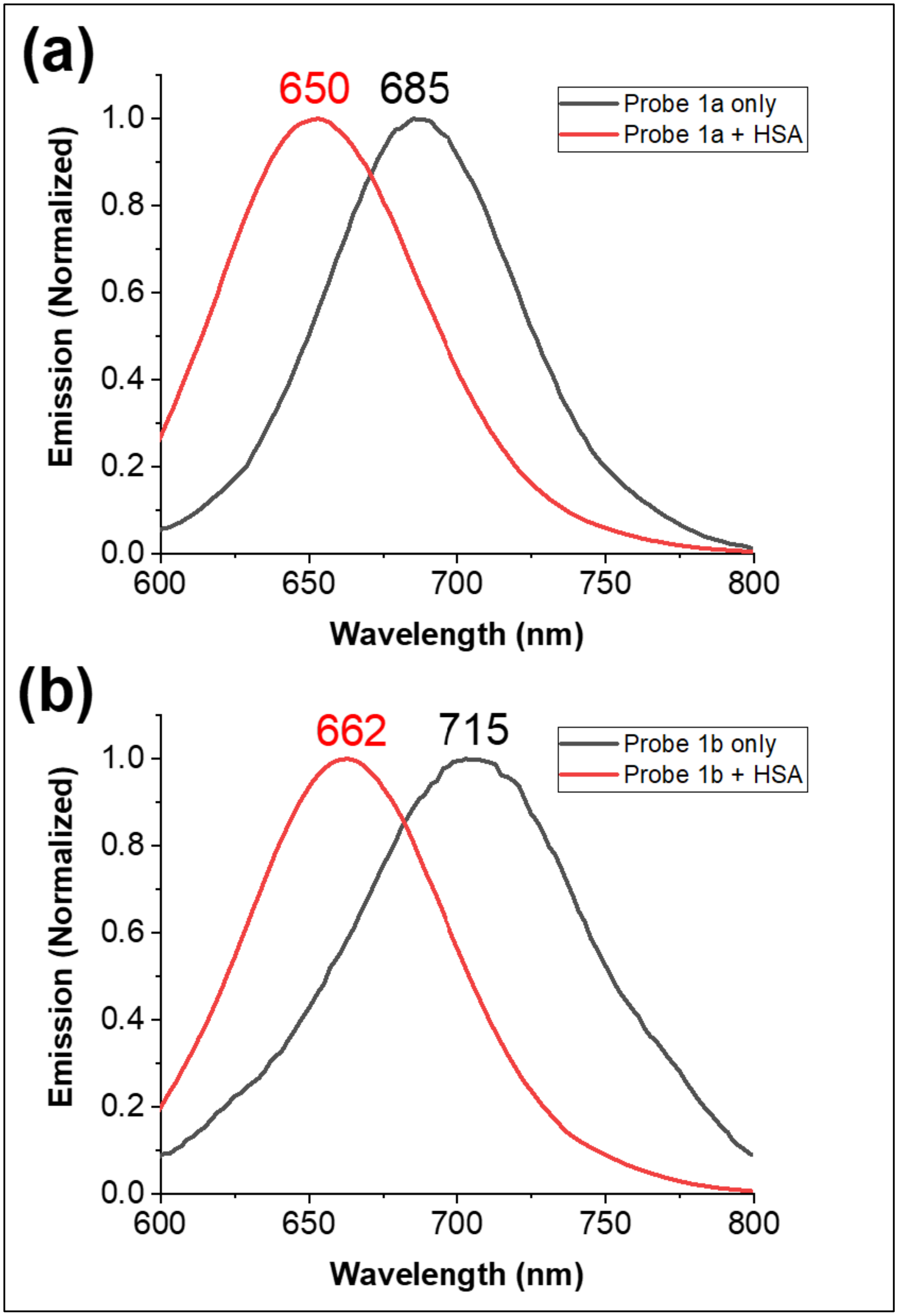

Figure S4.6 The normalized emission recorded for probes **1a** (a) and **1b** (b) in the absence (grey line) and presence (red line) of 1 equivalence of HSA at room temperature

(Probe concentration: 1.0 x 10^{-6} M). Probes were excited at 520 nm and the emissions were collected from 550 nm to 800 nm,

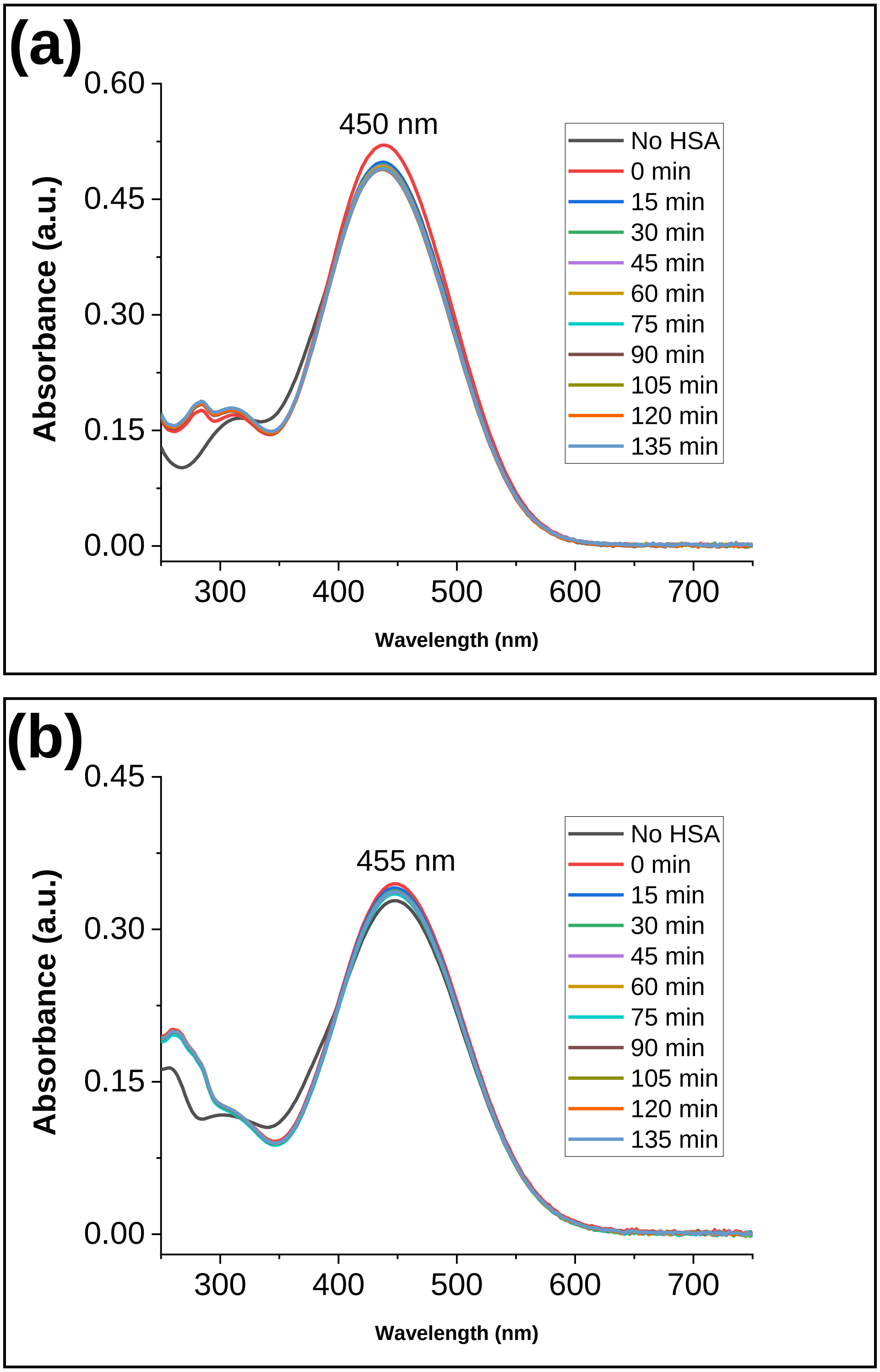

Figure S5.1 Absorbance spectra acquired for probe **1a** (a) and **1b** (b) (10 x 10^{-6} M) in the presence of HSA (0.5 mg/mL in water) over different time intervals at room temperature.

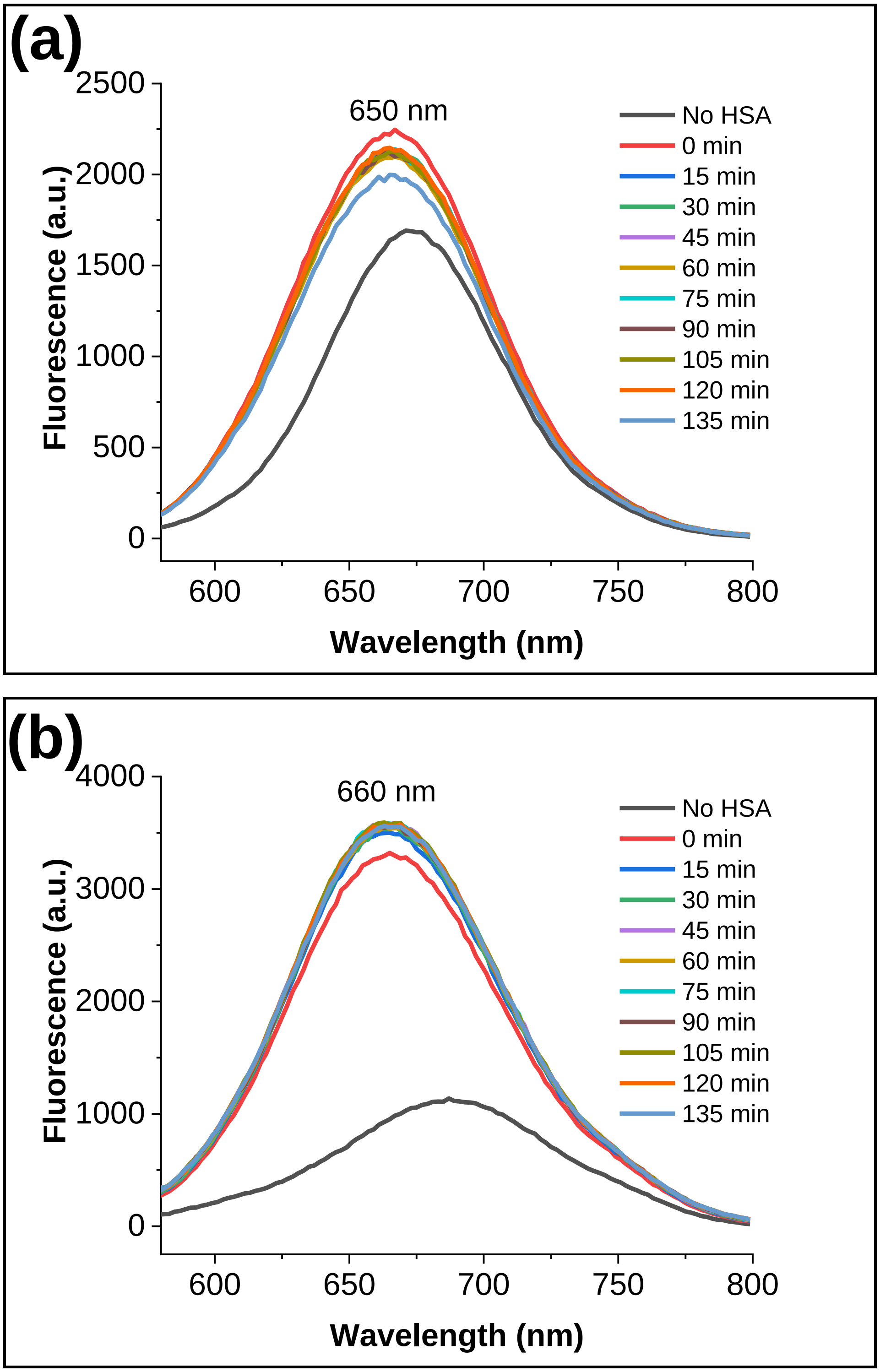

Figure S5.2 Emission spectra acquired for probe **1a** (a) and **1b** (b) (10×10^{-6} M) in the presence of HSA (0.5 mg/mL in water) over different time intervals at room temperature. Probes were excited at 500 nm and the emissions were collected from 530nm to 800 nm.

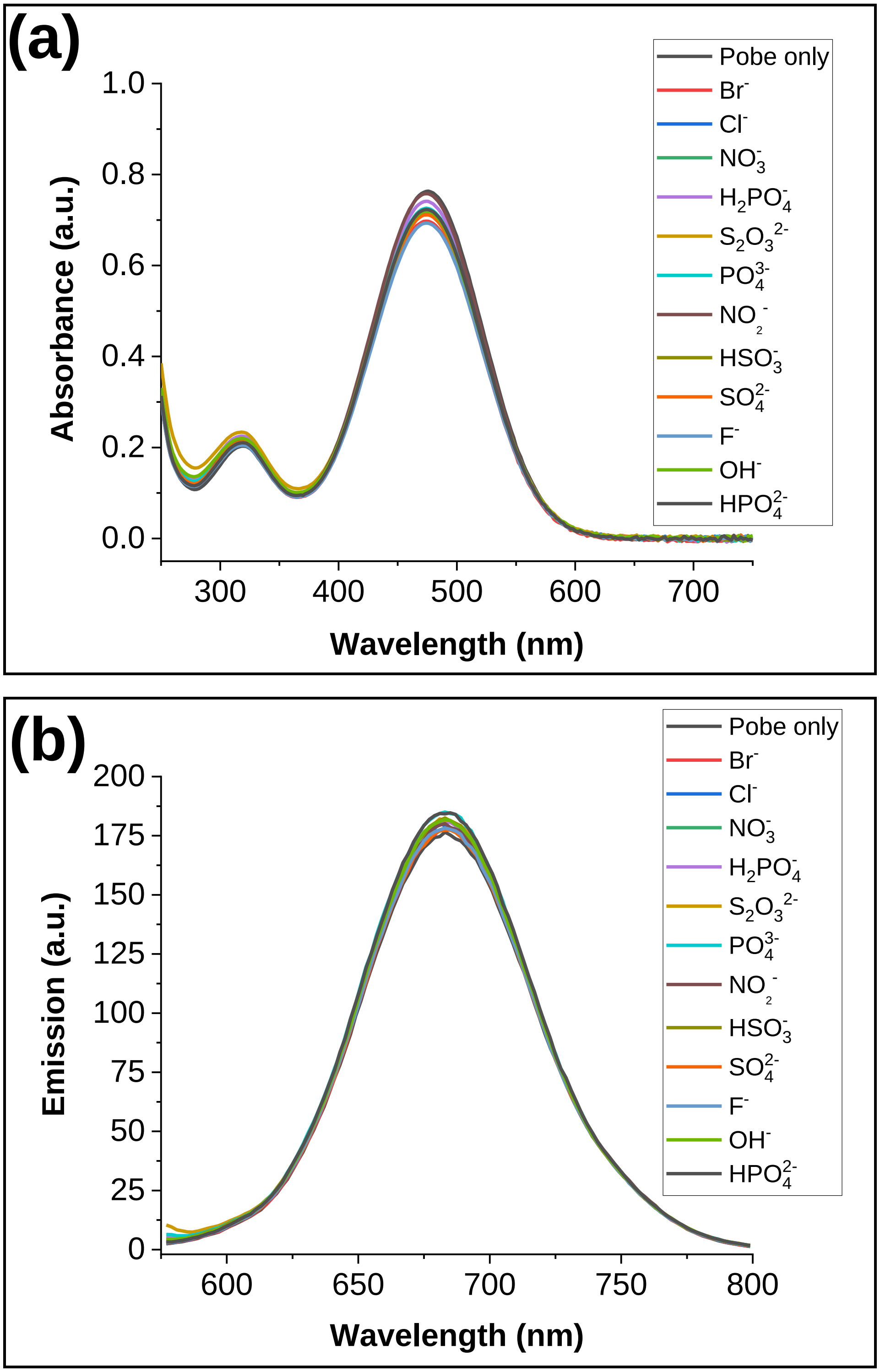

Figure S6.1 Absorbance (a) and emission (b) spectra acquired for probe **1a** (10×10^{-6} M) in water: acetonitrile (1:1) with the addition of various anionic species (10 equivalence) at

room temperature. Probe was excited at 500 nm and the emissions were collected from 530nm to 800 nm.

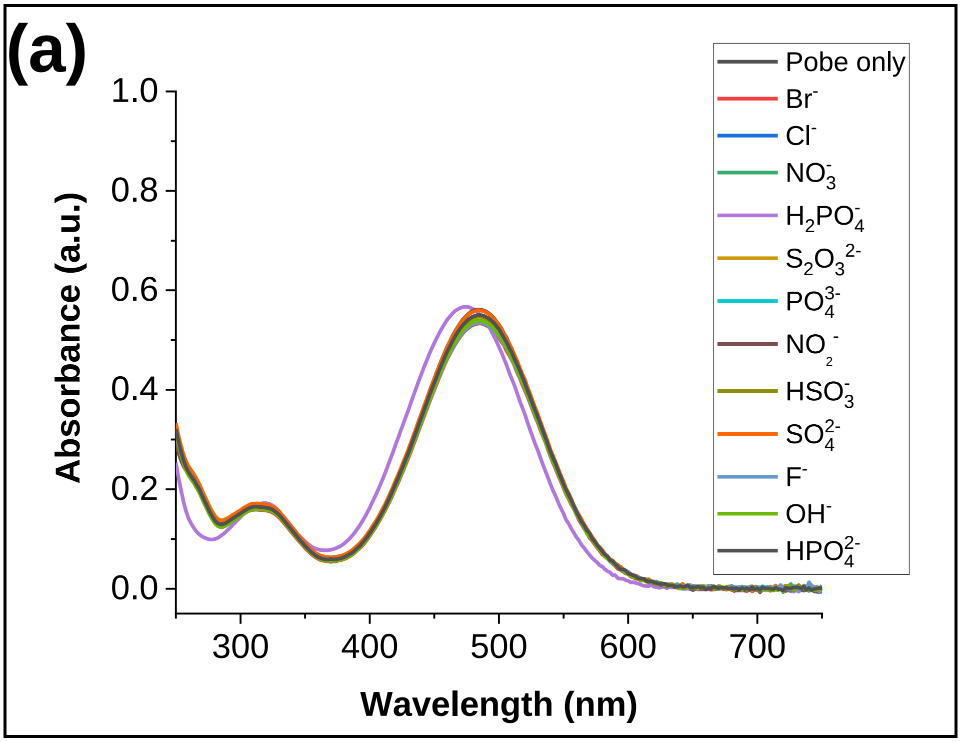

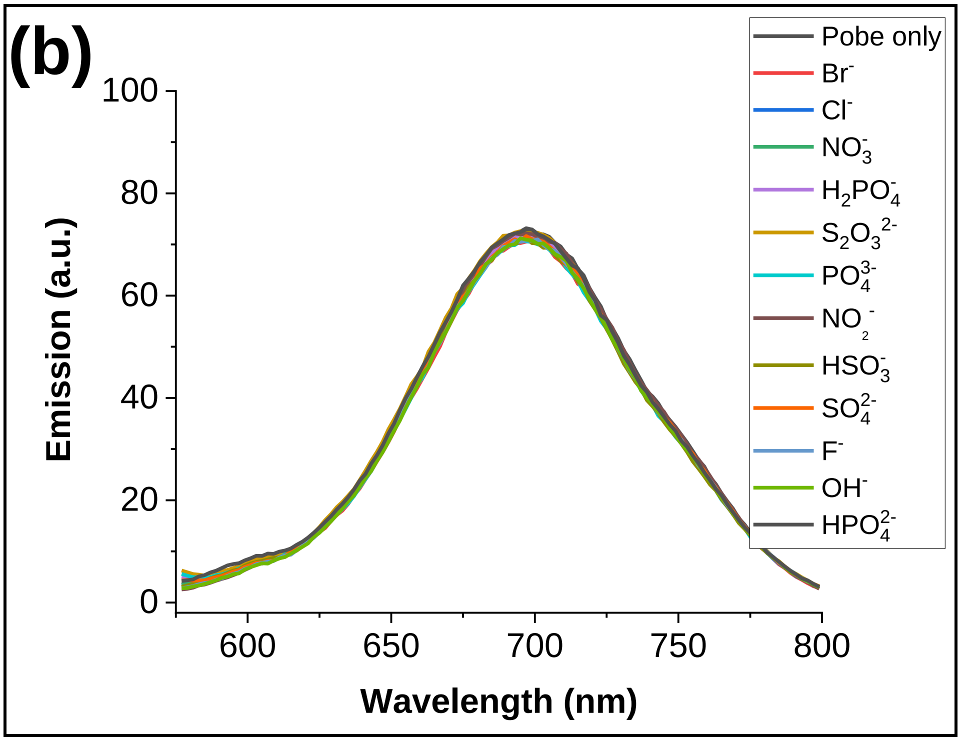

Figure S6.2 Absorbance (a) and emission (b) spectra acquired for probe **1b** (10×10^{-6} M) in water: acetonitrile (1:1) with the addition of various anionic species (10 equivalence) at

room temperature. Probe was excited at 500 nm and the emissions were collected from 530nm to 800 nm.

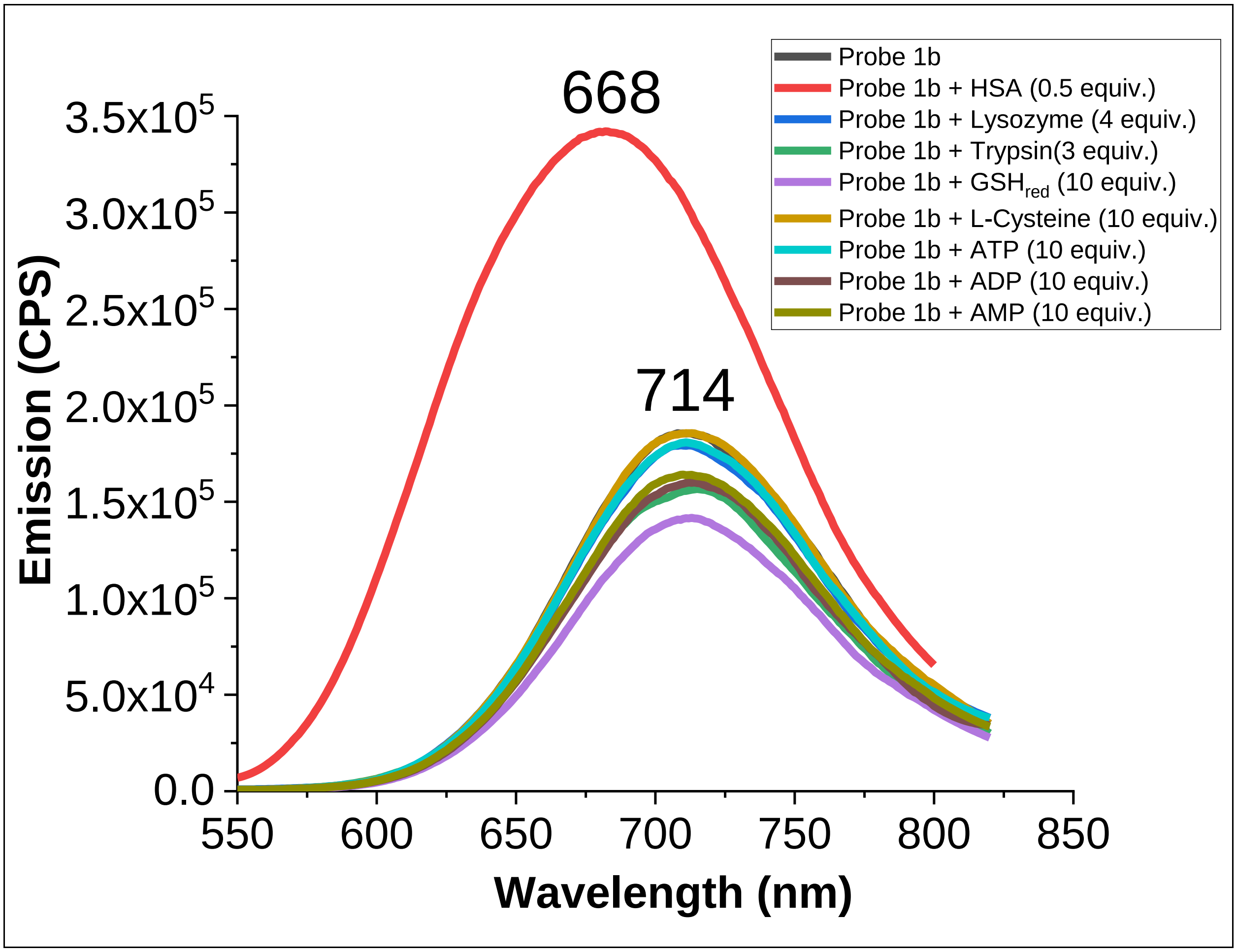

Figure S6.3 Emission spectra acquired for probe **1b** (10 x 10^{-6} M) in water upon addition of different biologically important species at room temperature. Probe was excited at 520 nm and the emissions were collected from 540nm to 800 nm.

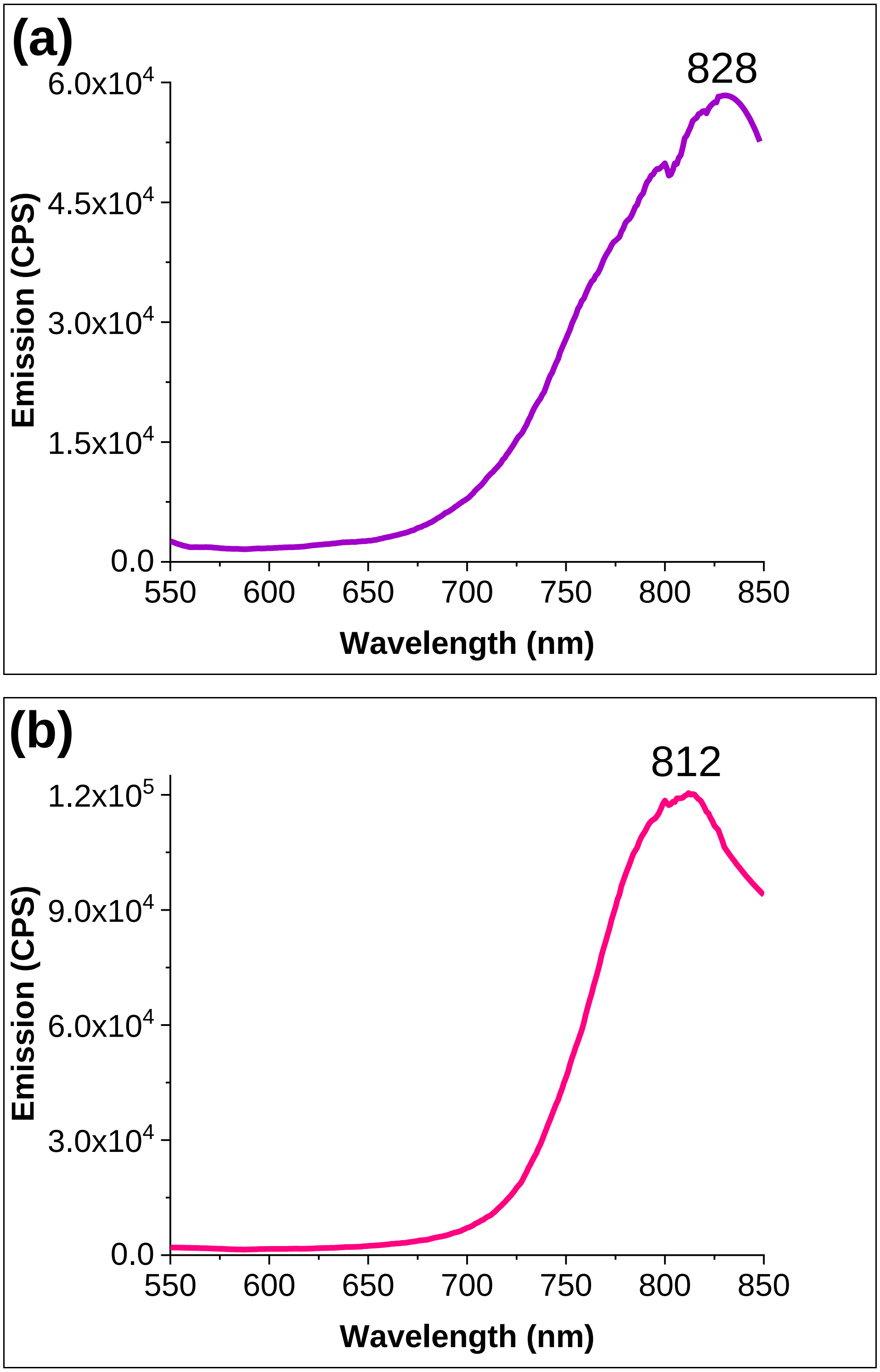

Figure S7 Solid-state fluorescence spectra acquired for probes **1a** and **1b** at room temperature. Both probes were excited at 520 nm and the emissions were collected form 550 nm to 850 nm.

Table S1.1 Limit of detection (LOD) and limit of quantification (LOQ) calculation for 1a with HSA

SUMMARY OUTPUT

Regression Statistics	
Multiple R	0.993976
R Square	0.987988
Adjusted R Square	0.986653
Standard Error	0.188422
Observations	11

ANOVA

	df	*SS*	*MS*	*F*	*Significance F*
Regression	1	26.28095	26.28095	740.2506	5.93E-10
Residual	9	0.319525	0.035503		
Total	10	26.60047			

	Coefficients	*Standard Error*	*t Stat*	*P-value*	*Lower 95%*	*Upper 95%*	*Lower 95.0%*	*Upper 95.0%*
Intercept	0.056001	0.106284	0.526902	0.611	-0.18443	0.296433	-0.18443	0.296433
X Variable 1	19.55168	0.718613	27.20755	5.93E-10	17.92607	21.1773	17.92607	21.1773

SE of Intercept	0.106284
SD of Intercept	0.352505
**LOD**	0.059496
**LOQ**	0.180291

Table S1.2 Limit of detection (LOD) and limit of quantification (LOQ) calculation for 1b with HSA

SUMMARY OUTPUT

Regression Statistics	
Multiple R	0.996892
R Square	0.993794
Adjusted R Square	0.993105
Standard Error	0.45985
Observations	11

ANOVA

	df	*SS*	*MS*	*F*	*Significance F*
Regression	1	304.7769	304.7769	1441.287	3.03E-11
Residual	9	1.903154	0.211462		
Total	10	306.6801			

	Coefficients	*Standard Error*	*t Stat*	*P-value*	*Lower 95%*	*Upper 95%*	*Lower 95.0%*	*Upper 95.0%*
Intercept	0.023395	0.25939	0.090192	0.93011	-0.56339	0.610176	-0.56339	0.610176
X Variable 1	66.58167	1.753797	37.96429	3.03E-11	62.6143	70.54903	62.6143	70.54903

SE of Intercept	0.25939
SD of Intercept	0.8603
**LOD**	0.042639
**LOQ**	0.129209
